# Supplementary material for: ST3GAL1 is a target of the SOX2-GLI1 transcriptional complex and promotes melanoma metastasis through AXL
Source: Nat Commun. 2020 Nov 17;11:5865. doi: 10.1038/s41467-020-19575-2 (PMC7673140; doi:10.1038/s41467-020-19575-2)
Supplement: Supplementary file 1 — Supplementary Information [file 41467_2020_19575_MOESM1_ESM.pdf]

SUPPLEMENTARY INFORMATION

ST3GAL1 is a target of the SOX2-GLI1 transcriptional complex and promotes melanoma metastasis through AXL

Pietrobono et al.

SUPPLEMENTARY FIGURES

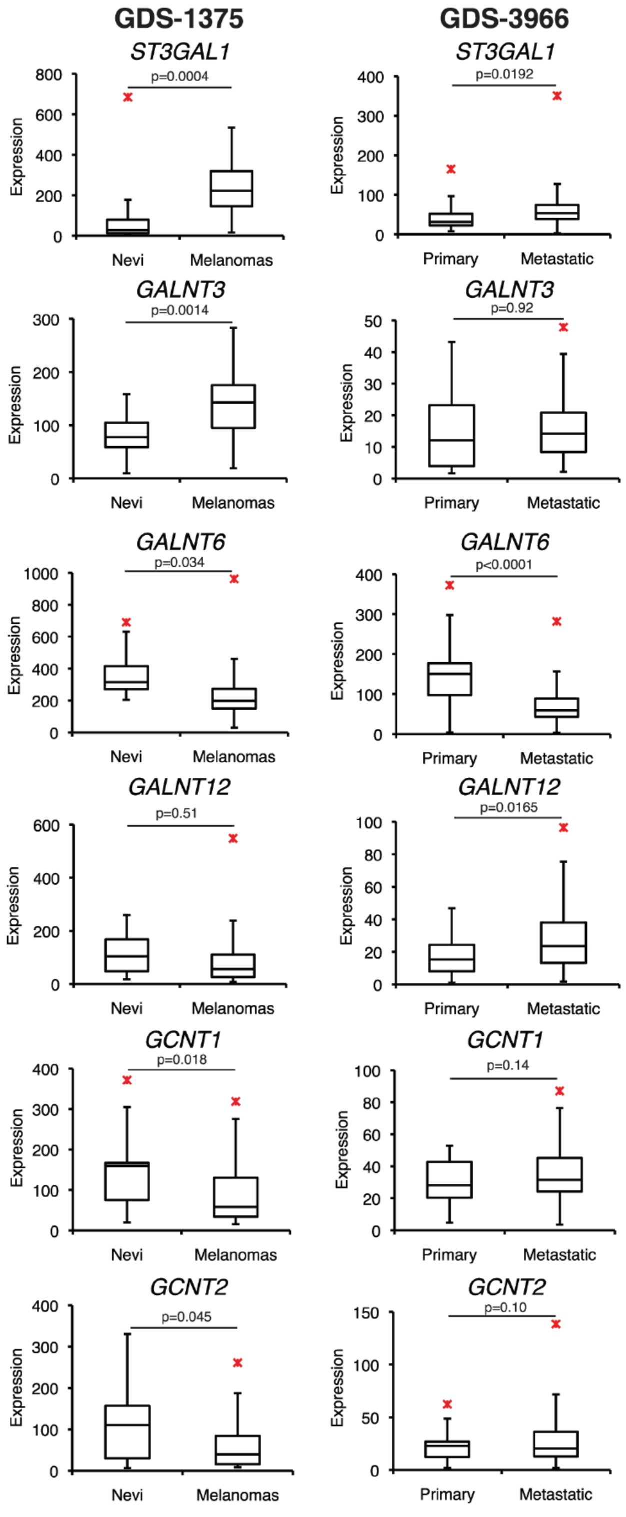

Supplementary Fig. 1. Expression of the 6 glycosyltransferases identified in the transcriptome analysis of SOX2- and GLI1-silenced cells. Box plots illustrating expression of *ST3GAL1*, *GALNT3*, *GALNT6*, *GALNT12*, *GCNT1* and *GCNT2* mRNA in melanoma samples compared to nevi and in metastatic compared to primary melanomas. Box-plots report median (central lines), 25th and 75th percentiles (box limits), and upper and lower whiskers represent values no further than x1.5 interquartile range (IQR). Lines above and below the box show the minimum and maximum. Outliners are represented with red crosses. Data were obtained from the analysis of public microarray datasets from two independent clinical cohorts. In GDS1375: nevi (n=17), melanomas (n=45). In GDS3966: primary melanoma (n=31), metastatic melanomas (n=52). *P* value was calculated by two-tailed unpaired Student's *t*-test.

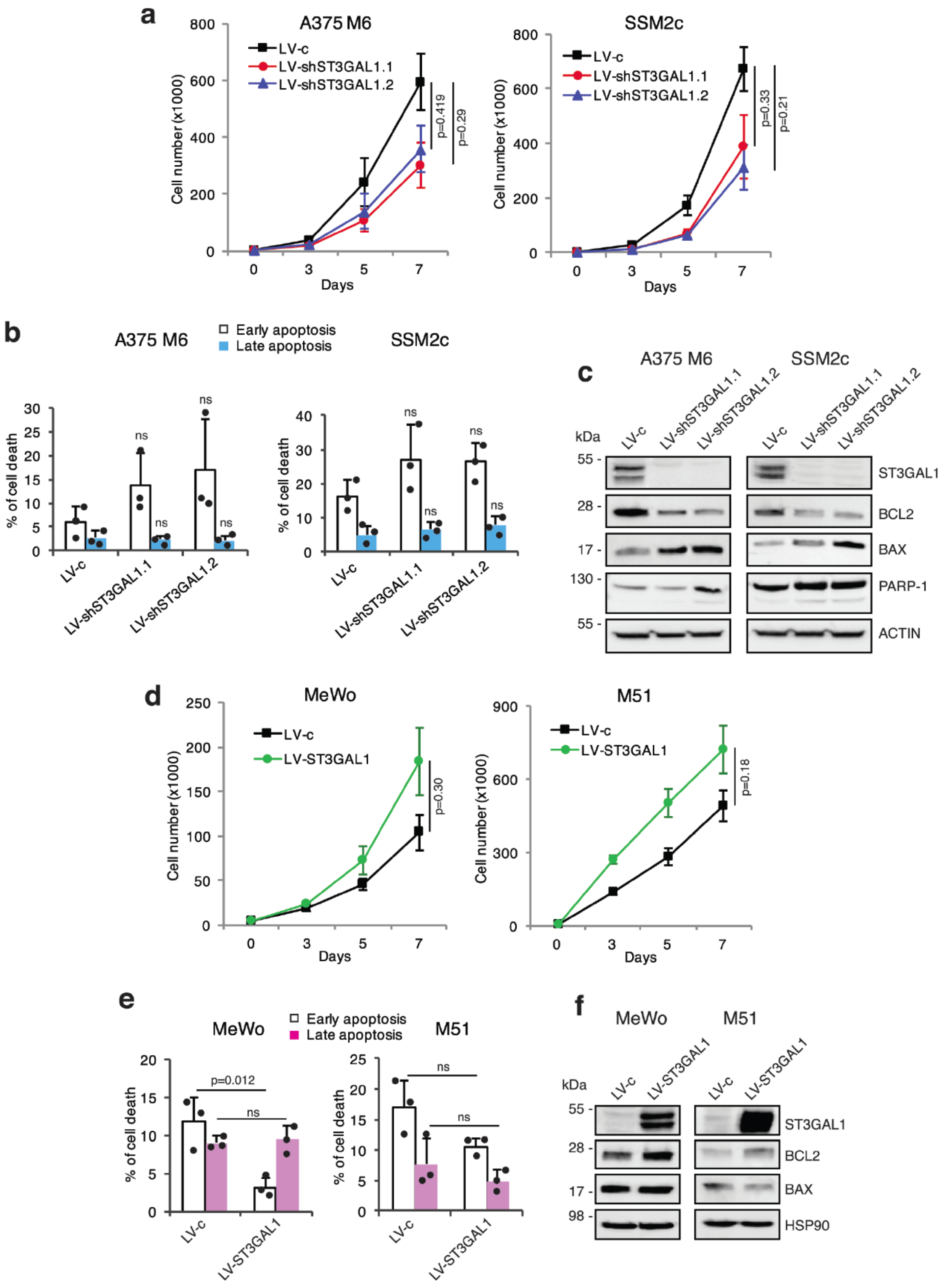

Supplementary Fig. 2. Modulation of ST3GAL1 does not significantly affect melanoma cell growth. a Growth curves of A375 M6 and SSM2c cells transduced with LV-c, LV-shST3GAL1.1 or LV-shST3GAL1.2. Data are expressed as mean ± s.e.m. *P* value was calculated by two-tailed unpaired Student's *t*-test (n=3 biological independent experiments). **b** Percentage of early (Annexin V+/7-AAD-) and late (Annexin V+/7-AAD+) apoptotic cells in A375 M6 and SSM2c cells transduced

as indicated. Data are expressed as mean ± s.d. *P* value was calculated by two-tailed unpaired Student's *t*-test (each LV-shST3GAL1 vs LV-c) of n=3 biological independent experiments; ns, not significant. **c** Western blot of ST3GAL1, BCL2, BAX and PARP-1 in A375 M6 and SSM2c cells transduced as indicated. **d** Growth curves of MeWo and M51 cells transduced with LV-c or LV-ST3GAL1. Data are expressed as mean ± s.e.m. *P* value was calculated by two-tailed unpaired Student's *t*-test (n=3 biological independent experiments). **e** Percentage of early (Annexin V+/7-AAD-) and late (Annexin V+/7-AAD+) apoptotic cells in MeWo and M51 cells transduced as indicated. Data are expressed as mean ± s.d. *P* value was calculated by two-tailed unpaired Student's *t*-test (n=3 biological independent experiments); ns, not significant. **f** Western blots of ST3GAL1, BCL2 and BAX in MeWo and M51 cells transduced as indicated. Blots in (**c**) and (**f**) are representative of n=3 biological independent experiments. ACTIN and HSP90 were used as loading control. FACS sorting strategies used for apoptosis analysis are shown in Supplementary Fig. 13. Source data are provided as Source Data file.

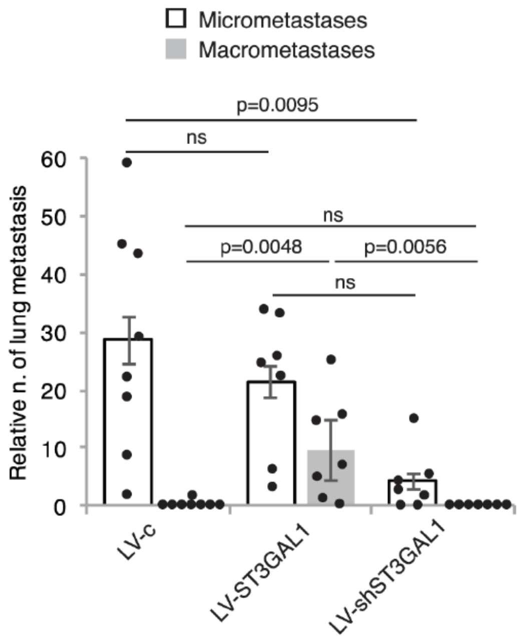

Supplementary Fig. 3. ST3GAL1 overexpression increases the number of macrometastases in vivo. Quantification of lung micro and macrometastases in athymic nude mice. A375 M6 melanoma cells were transduced with LV-c, LV-ST3GAL1 or LV-shST3GAL1.1 and injected subcutaneously into the flank of athymic nude mice (n=8 per group). See Figure 5a for the schematic representation of the in vivo xenograft experiment. Graph shows quantification of micro and macrometastases in the lungs. Data represent mean ± s.e.m. *P* value was calculated by ANOVA and Tukey's test; ns, not significant.

Supplementary Fig. 4: Sequence of the *ST3GAL1* enhancer II.

UCSC Genome Browser GRCh37/hg19
>hg19_dna range=chr8:133579601-133584401 5'pad=0 3'pad=0 strand=+ repeatMasking=none
Position: from -12461 to -7661 bp from TSS (see Fig. 6A for details).

CACTGCAGCATTCGGGCGGAGGCCCCCACACTCTTCAAGGCAGCTTCCAGTCAATAACTCAACATGGCATGGGCATCCAGGCTTG
GCCATTTCTGCCCAGCATGCCCACCTTCTGTGACAATCTTTGTGCTGGAGCTACCCATTGGCTGACATTTTCTCAGAGCTGCAGTTA
GAGGATCCTTCTAGCCAATCCTCCTTCCTCTTCCTTCTGCCTCATTTTACAGGTGTCAAAGTCACATCTAAACTTGTGGTCTTAAGGC
ATCTCATTTGCACTTCTGCCCCCTTTCCTCCTTCTTCCTTAGGCATTACCCCCCAGTAAATCTCTTGCAGTTCTAACTTCTTGGTGTC
TGTTTCCTGGAGCATCCAAATGCATCAGCCTCCTCAGGGTCCTTCCAGCTTGACTGCTCCCTGGCCCCCAGCAATAAGCATATGTG
TTAGGTTTTTGTTTTGGTAAAGCCCCATCTCCAGATGCCAAATATGTTGTGATTGCTATTGCTACCAAAAGAAAAATTACCCCCAAAA
TTTAGGAAATTAAAACCATTGTATTTCTCCCACAATTTTGTGGGTTCCATTTTGGGAAAGCTCGGCAGGATAGTTTTCACTTGGGGTC
TCTCCTGCCGTTGCAGCCAGATGTTGGCTAGAACTGCAGTAATCTAAAGGCTTGACTGGACTGGATATCAAAGATGGCAGACCTGT
CTGTCTGGAAGTTGATGCGGTAGCCAGGAACACTGCTGAGGCTATGATTGGAACGCCCCTGTCTGGCATTTCCAGCATGGCAGTCT
CAGAGTGGTCGAACTTCTTACATGGTGGCTGGATTTGCCCAGAGCAAGTGTCCCAAGAGAGCCAAGCAGAATTGGTATGGCTATGA
CAGCCATCTAGTGGTATGGCCGTGAAAGCCTTATGGCATCATTTCTATAGGTTAGAAGCAAGTCACCAAGTTTAGCCCAGATTCAAG
GTAAGAGGGCAGAGACTCCACCTCTTGATGGAAGGAGTGTCCGGTAATTTGCAGATGTTTTCAGACTTTCACAGTGACAAATACGA
CAAAAGTAGAAGCCAACACTTAGTCCCATATGCAGCCACTTAAAGGCCCTATAGGACCAGCCAGCTACCTGGTGGCAAGTTGATTA
CACAATAACAATTCTATTATGGAAGAGAAAGACCCTTATTTTCACTGGAAAATGCACTTATTCTGAACATAAATCTTTCTTCCTTCCCT
ATAATGCTTCTGCCAAAACCACTATCTGTGGACATACCTAACAAATGCCGTATTCACTGTCATAGTATTTCATGGAGTGTCACTTCTA
ACCAAGCAACTCAATTTCCAGCAAATGAAGCATGCCAATAGAATGATGCCATAGAATTCAATGAACTTACCTGGAGCAGCTGGCCTG
ACAGAACAATGAAGTGGCCTTTTGAAGGTTTAATTAAAGTGCCAGGATGTGATATAATGCTTTTTGTGGATGAGGTAATGTTCCTTTG
GATATAATATAGAATCTGAATCCCCATCCAATATATGGTGCTGTTTCTTTCATAGCCAAGATGCATAGATCTGGGACCAAGGGATGG
GAGTGAGTATGGCCCACTCACTACTGCCCCTACTAATGAACTCGACAACATTTTACTTCCTGCTTCTGGAACTTGGGGCTCTGCTGG
TTTAGAGAGATTGGTTCCAAAGGAAAGAGTGCTTTCATCAGAAAACACAACAATGCTGCCTTCTAATTGCAAGTGGAGACAAATCTA
GCCATGTTGGGGTCTTTGTGCCAGTGAACTAACAGGCAAAGAAAGGAAGTTAGTGTACTGGCTGGGAGATTTCACCATGACAAGAA
AATGAATAGCTACTGCTCAAGGGCAGTAGGAGAGAAGAGGGAGGGATTTCACTGAAATGCAGATAATTCTTGGGGCATCTTTTTAG
TACCTCCAGTTTTGTGGTAACAGTAAATGGAAAACTAAACCACCCAATATAGATAGGACTGTCAATGGCACAGAATGTGCAGGAATG
AAGGTTGGGTTGCCCCATGACATCAGGAACTACCCCGTGGAGGTGCTTGCTGAAGGAAAGGTAACATGAAATAAGTAGTGGAAGG
AGAAGGCTATAAATATCGGTTATCAGCTGCAGCCACATGATCAGCTTCAAAAACAAGATCTATAGTAGTGATGGTATTTCTTCCTTGC
TTCGATATGAATATATATGTGTGTAAACACACATATCCATATACAAAGATCTGTCTAGTTTTTGGAACACTCTTCCCTCAGACATCTTC
CTGGTTTACTACCTGACAGCTGGATCTCCTTGGGGAAGAACCCTGCTATACTGCCAAAATTTTATTCTATAAATCTTCCTTTTAGCTT
TCTGCAAAGGGGCCCACAATTACGAGGGTGACTGTGCACTGAGAGGAAAGAAACACCCAGACATTTCAGGGATTACTGGACATTG
GCTCTGAACCAATGGAAATTCCTGGGGACCCAAAACAACACCATGGTCCACAGTTAGAGTGGACCACATATGTACATCCACATATG
AATTTATCTATATACGTAAATCCCTTCACTTTGTTTCTTTTTTTTTCCATTCTAATATAAGATGTGTTAACAGTGGTTAACTTTATATCTC
CATATTTAATTCATAGGATATAAAAATGAGAGTGTGAGTCCTCTAGAAGAGAAATAACCATCAGTCACGGATGAAAGAGGTCACATG
CAGGACTCTGTGTATCTTTTTTGGGAGAGGGATAATGTCCTCATCTGCTTGGGCTGCCACAAAAAAGTACCACAGACTGGGTGCGT
AAAACAACAGAAATGTATTTCTCCCAGTTCTGGAGGCCAAAGTCCAAGATCTCCTCCTGACTTAGTGCTATGTCCTCAGATGGCCAG
ATGGCTGAGAGAGAGTGGTCTCCAGTGTCTCTTCCTCTTAAAAACACAAATACCATCATGAGGAACCAACCCTCATGATATTATTTAA
TCCTAATTATCTCCCAAAGGCCCACCTCCAAATACCATCACAATGGGGGTTAGGGCTTCCACATATGGATTTTGTGAGTACACAATT
ATTCAGTCCATAACAGATGGCACAGTTTTGTTTGTTTAATGGATAATTCTATTAGTTTGGTCAAAAGTATAATTTTGCTTTCTCTCTATG
TGGAAATTAAATACAGTCCTGACAAATGTACATGATTTCCAAGTTGGTAGAGGTTGAGTTCTGTTGGATTATGATATGTCAAGTTGGC
TAAGGCTGGGAGCTGTTTTCCAGAATTTCCTTCCCTACGTGGTTCCTGGGTAGAGTTGACCATGAAGAAAGGTGTTATACTCAGTCA
TTGCCATCAGTCGCAGTGATGGACAGATGCAGAGGGACCCAGCCTGCTGCAGCTGATCCTTGATCTCCACCACTGTGTGTCCATCC
CATCTTTCTTACCGCAGGCCTGCTGACGAAGGCTGGCTCCAAGCCCACCCACCAGGATCTTGGCCACGGACCAGAAGGGTAGCAG
ATAAATAGAGGCAACAATTTCAGCAGCATGATAATTTCCATAGACCTCTCCACAAGCTCCCAATCACAGTCCTACTTCAGCTTCTCAG
ATTCTGTGCAAGTTCTGAATTGTCTACCTGTACCAGTGCTTCTTCTTCCAATTCTCTACCTCTCCCTTCTAGAGCTTCACTACCCAGC
TCTTCCCACATTTGTGCCCAATTGCATTCCTATAACCAGTCCCTCATTCTCATCATATTTTTTTCTAACTGATGCATTTTGGTTATCTG
CTAAACATTAGCAGAGTCTTCCACTGGAAGCCTGTTTAATAGAGAAAGGCCCTGTCTGAAAAAGCACCCATCACCCTTGATTCCCTC
ACTCTGACTCTCCTTCACAGCTCTGAACACCACCTGACATAGTCTGTGTGTATGTATTTGTCTGCTTTGTTACTACTAAATCCCTGAT
GAACAGAGAGGGGGCTAGCACTCAATAGTTGCTCAATAAGTATTTGTTGAATGAATATTTGGGGAAACTGACTTAAATGTTGCTATAT
CATGATCAGAGTAACAGTAATTTGTCCTACAATGTATGTGAAATATTGAATTCATAATGTTCTAGATAAAGAAGAGGTAGGATATTAAA
AAAGTAAAGAAGGAAATAGAGGAGAAGGAGTGGGATGGGGAGTAAAAAGGTGAAGAAAAAGAATAAGGGGAGAAAAGTAATTGGG
GAGGGAGGGGAAGAAGGAGCAGGGAGGAGGAGAGACAGGAGAAAGGGAGGAGAAGAGAGAGGAGAAAGGGAAGAAGGAGAGA
GAGAAGAGGCTCTAAATGTATGGCTGACACTGTACTAAGTGCTTTCTGCAGTTTATCTTGAGACAAACCTGTGAGGCAAGTGTTATT
ATTCCCAGATTATACATGAAGCCACTAAGGCTCAGAGAATTAAAGGACTTGCTGAAGGTCTTTACAAAGTGATTGAGCTGGAATCCA
AATATAGAGTGTCTGGTTTCAAAACTCATATTCTTGCCATTATACCATGCCACCAGAGAAAGCAGACACATCATGGGAAGGAAAATG
GAAATGCTGTTTAAAATCATATTAAAGTATTCCAATTGGGTCTGAAATTACAGTTTTAACTTCTAAGCTTGACCACTTTGCTGAAGTAC
CAATTTAATACTCAGTCCTTTTATCTATGAATTAATACTTTGCATAAGTAGACAGTATTTATAATCAGTGTTTTATGCAATAGTTCTATG
ATCTTACTAAACATTTTCAATACCAACCCTTTATTATAGGTAAATGATGAGTTATAGCATTTAAGACATACATTTTAATTTTAAGCTATC
TTAAG

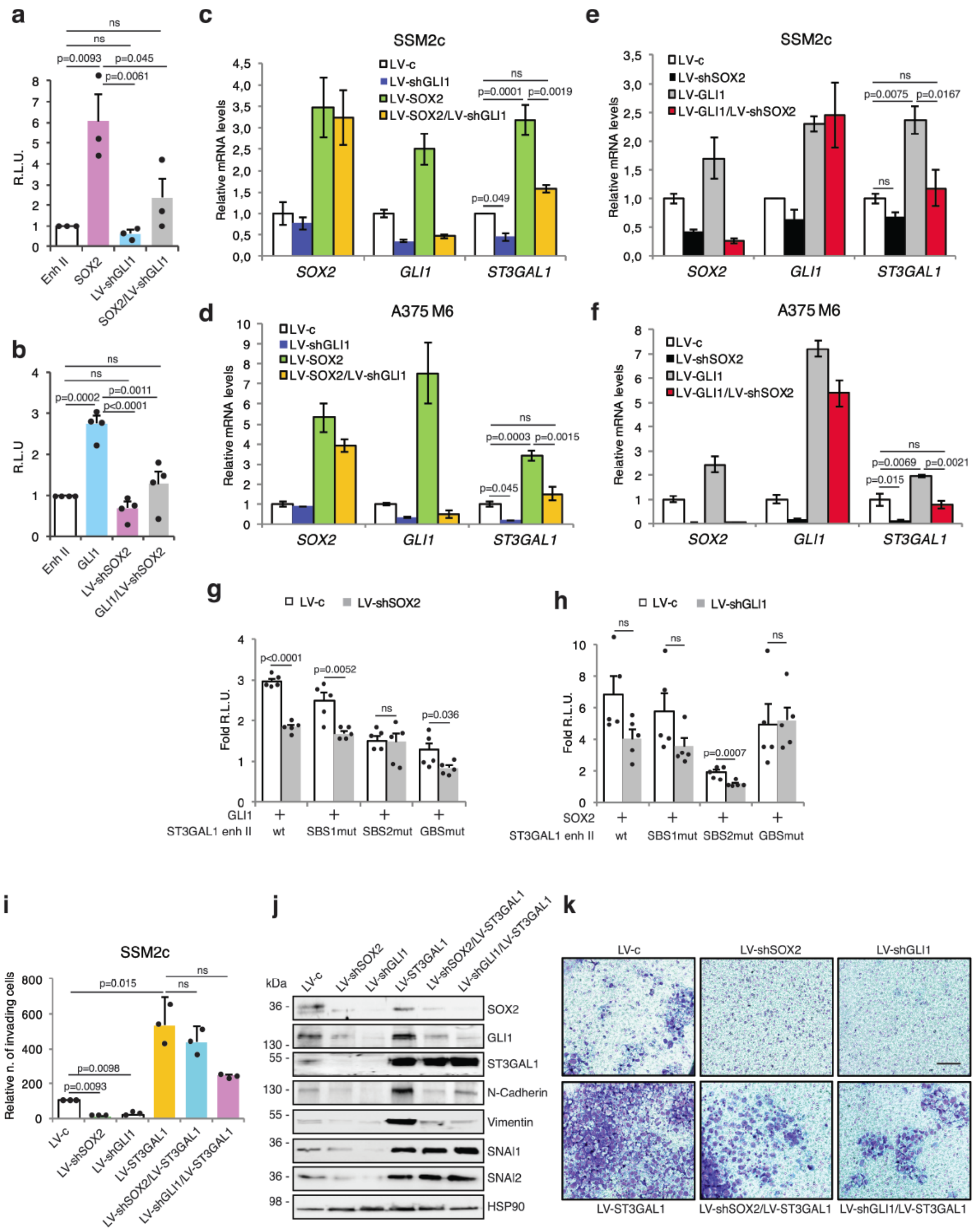

Supplementary Fig. 5. ST3GAL1 is a target of SOX2 and GLI1, and it rescues the effect of SOX2 and GLI1 depletion on melanoma cell invasiveness in vitro. a, b Quantification of dual-luciferase reporter assay after ectopic expression of SOX2 in SSM2c melanoma cells silenced for GLI1 (**a**) and after ectopic expression of GLI1 in SSM2c melanoma cells silenced for SOX2 (**b**). Relative luciferase activities of *ST3GAL1* enhancer II were firefly/Renilla ratios, with the level induced by the control equated to 1. Data are represented as mean ± s.e.m. *P* value was calculated by ANOVA and Tukey's test (n=3 biological independent experiments in **a**; n=4

biological independent experiments in **b**). **c-f** Quantitative real-time PCR (qPCR) analysis of *SOX2, GLI1* and *ST3GAL1* in SSM2c and A375 M6 cells transduced with LV-c, LV-SOX2, LV-shGLI1 or their combination (**c, d**) or with LV-c, LV-GLI1, LV-shSOX2 or their combination (**e, f**). LV-c was equated to 1. Data are represented as mean ± s.e.m. of n=3 biological independent experiments. *P* value was calculated by ANOVA and Tukey's test. ns, not significant. **g, h** Quantification of dual-luciferase reporter assay in SSM2c melanoma cells transduced with LV-c and LV-shSOX2 (**g**) or LV-shGLI1 (**h**), showing that while SOX2-induced transactivation of ST3GAL1 enhancer II occurs even in absence of a functional GBS (GBSmut), disruption of SBS2 reduces transactivation by GLI1. This effect is still abrogated when GLI1 is overexpressed in SOX2-depleted cells. See Fig. 6h for details. Data are represented as mean ± s.d. by two-tailed unpaired Student's *t*-test (n=5 biological independent experiments). **i** Matrigel invasion assay in SSM2c cells transduced as indicated. Data are represented as mean ± s.d. by two-sided Kruskal-Wallis and Dwass-Steel-Critchlow-Fligner method (n=3 biological independent experiments). **j** Western blots of SOX2, GLI1, ST3GAL1 and EMT markers in SSM2c cells transduced with LV-c, LV-shSOX2, LV-shGLI1, LV-ST3GAL1, LV-ST3GAL1/LV-shSOX2 or LV-ST3GAL1/LV-shGLI1. Blots are representative of n=3 biological independent experiments. HSP90 was used as loading control. **k** Representative images of (**i**). Source data are provided as Source Data file.

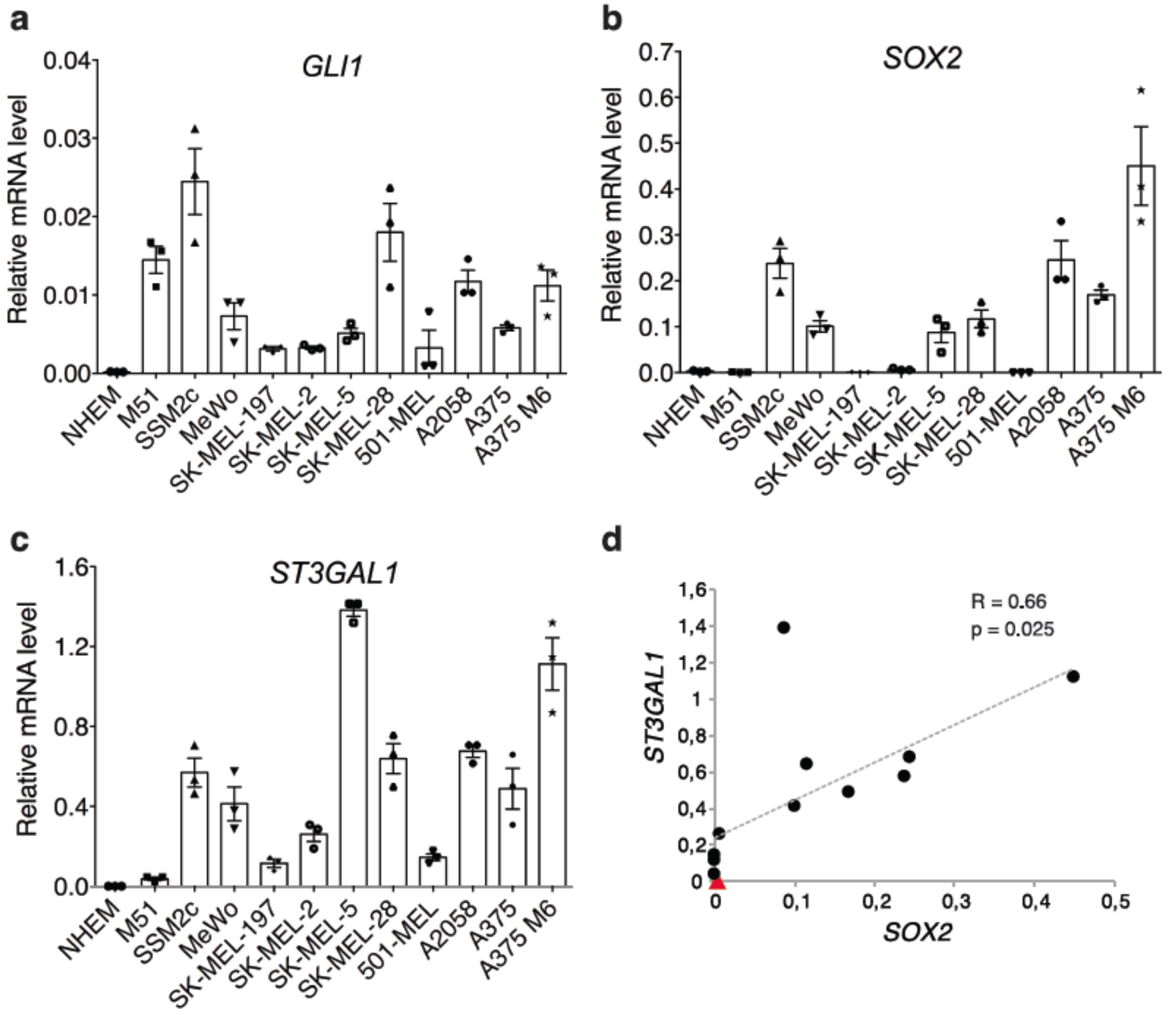

Supplementary Fig. 6. Expression of *GLI1, SOX2* and *ST3GAL1* in human melanoma cell lines. a-c Quantitative real-time PCR (qPCR) analysis of *GLI1* (**a**), *SOX2* (**b**) and *ST3GAL1* (**c**) in normal human epidermal melanocytes (NHEM) and in a panel of metastatic melanoma cells. Data are represented as mean ± s.e.m. of n=3 biological independent samples. **d** Correlation dot-plot of *SOX2* and *ST3GAL1* mRNA in NHEM (red triangle) and in a panel of metastatic melanoma cells (black circles) calculated using Pearson's correlation test (n=12).

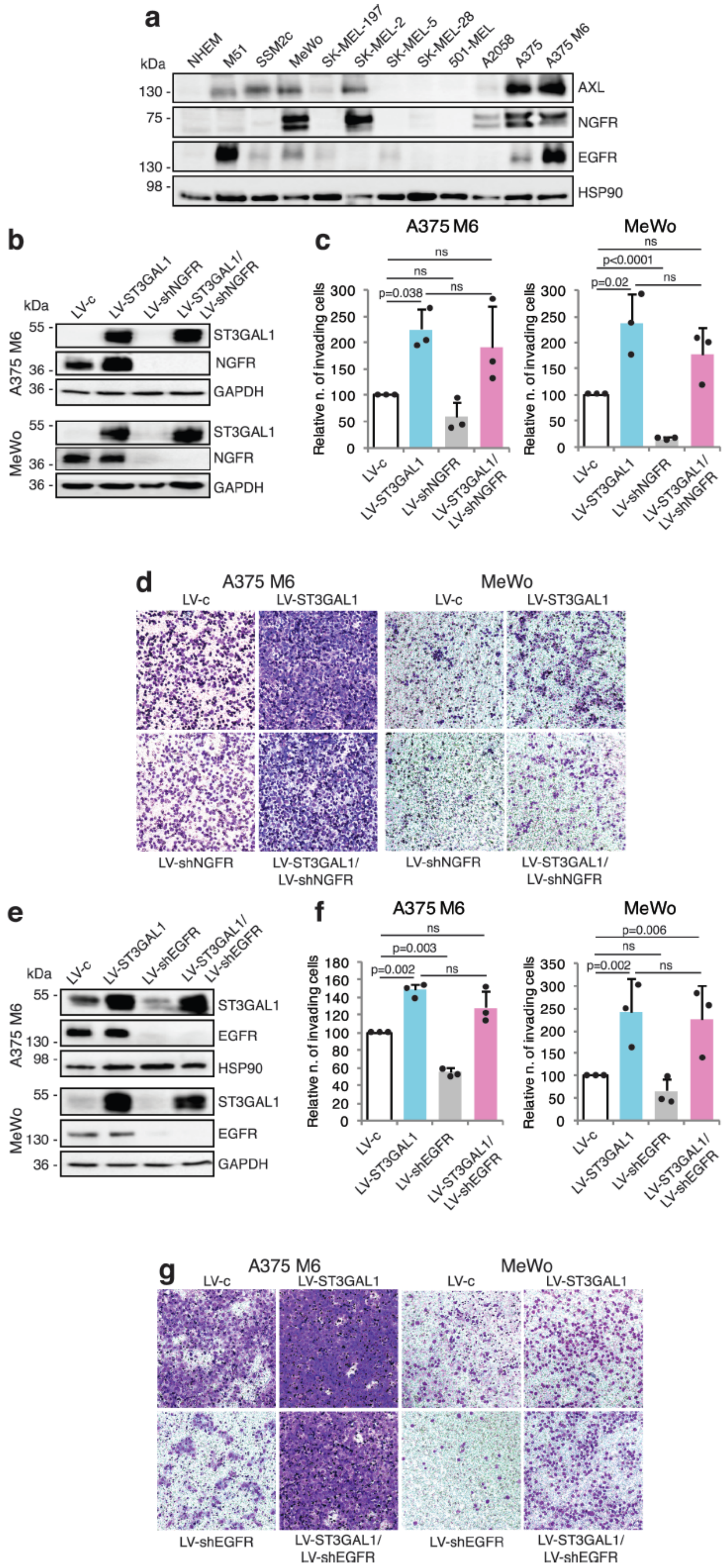

Supplementary Fig. 7. NGFR and EGFR are not the main mediators of the pro-invasive effects induced by ST3GAL1 in melanoma cells. a Western blots of AXL, NGFR and EGFR in normal human epidermal melanocytes (NHEM) and in a panel of metastatic melanoma cells**. b**

Western blots of ST3GAL1 and NGFR in A375 M6 and MeWo cells transduced as indicated. **c** Matrigel invasion assays in A375 M6 and MeWo cells transduced as indicated. Data are represented as mean ± s.d. *P* value was calculated using ANOVA and Tukey's test (n=3 biological independent experiments); ns, not significant. **d** Representative images of cells indicated in (**c**). **e** Western blots of ST3GAL1 and EGFR in A375 M6 and MeWo cells transduced as indicated. **f** Matrigel invasion assays in A375 M6 and MeWo cells transduced as indicated. Data are represented as mean ± s.d. *P* value was calculated by ANOVA and Tukey's test (n=3 biological independent experiments). ns, not significant. **g** Representative images of cells indicated in (**f**). Blots in (**a**), (**b**) and (**e**) are representative of n=3 biological independent experiments. HSP90 or GAPDH were used as loading control. Source data are provided as Source Data file.

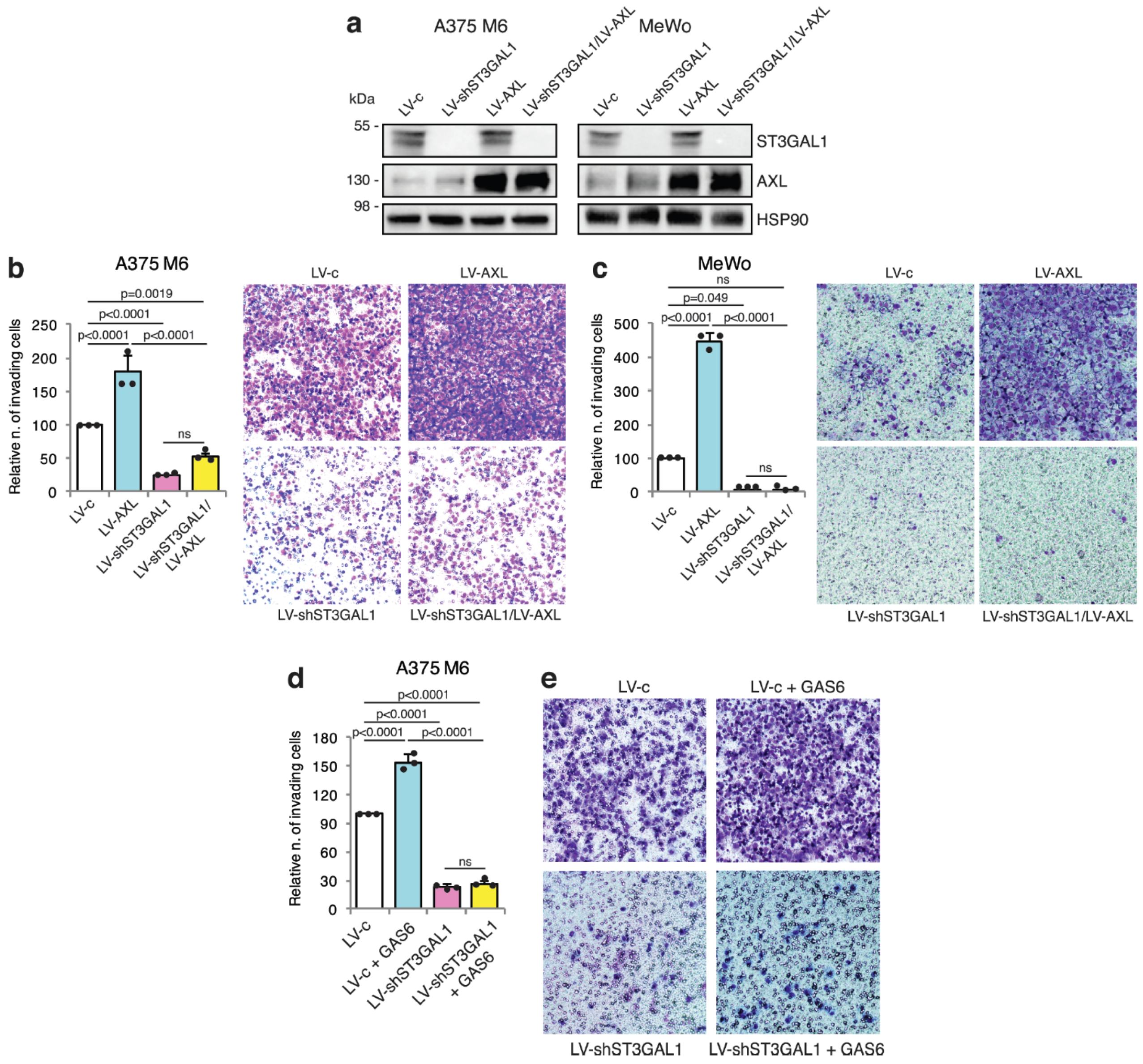

Supplementary Fig. 8. Ectopic expression of AXL or its activation by GAS6 do not rescue the effect of ST3GAL1 silencing in reducing melanoma cell invasion. a Western blots of ST3GAL1 and AXL in A375 M6 and MeWo cells transduced with LV-c, LV-shST3GAL1.1, LV-AXL or their combination. Blots are representative of n=3 biological independent experiments. HSP90 was used as loading control. **b, c** Matrigel invasion assays (left panel) and representative images (right panel) in A375 M6 (**b**) and MeWo (**c**) cells transduced as indicated. **d, e** Matrigel invasion assay (**d**) and representative images (**e**) of A375 M6 cells transduced with LV-c or LV-shST3GAL1.1 and stimulated with GAS6 (250ng/ml) for 24hrs. Data in (**b**), (**c**) and (**d**) are represented as mean ± s.d. and *P* value was calculated using Welch ANOVA and Tukey's test (n=3 biological independent experiments); ns, not significant. Source data are provided as Source Data file.

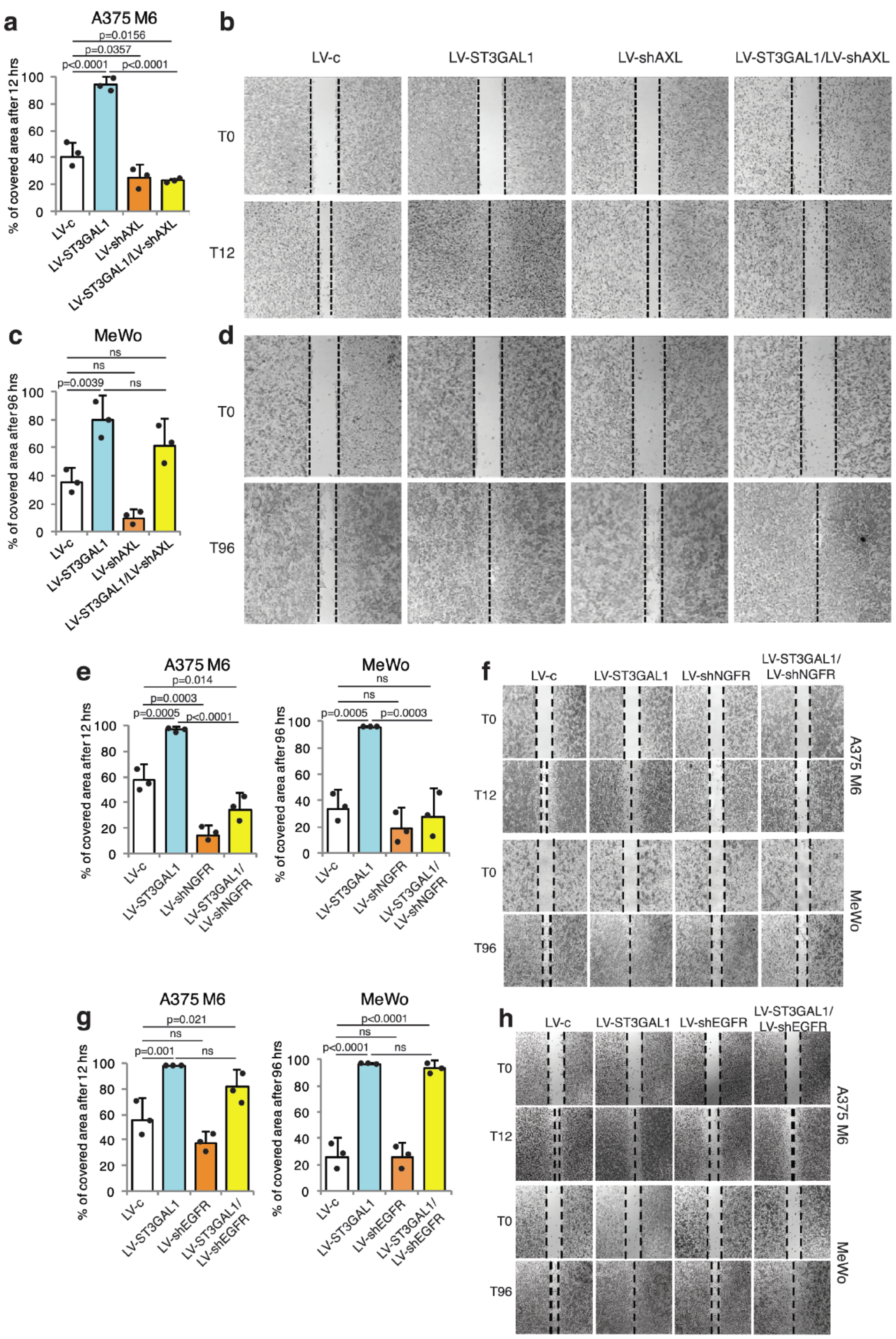

Supplementary Fig. 9. Effects of AXL, NGFR and EGFR silencing on the migratory ability of melanoma cells overexpressing ST3GAL1. a-d Wound closure (**a**, **c**) and representative images

(**b, d**) of A375 M6 (**a, b**) and MeWo (**c, d**) cells transduced as indicated. Data are represented as mean ± s.d. *P* value was calculated by ANOVA and Tukey's test (n=3 biological independent experiments). **e-h** Wound closure assays of A375 M6 and MeWo cells transduced as indicated. Data are represented as mean ± s.d. *P* value was calculated by ANOVA and Tukey's test (n=3 biological independent experiments); ns, not significant. **f, h** Representative images of A375 M6 and MeWo cells indicated in (**e**) and (**g**), respectively.

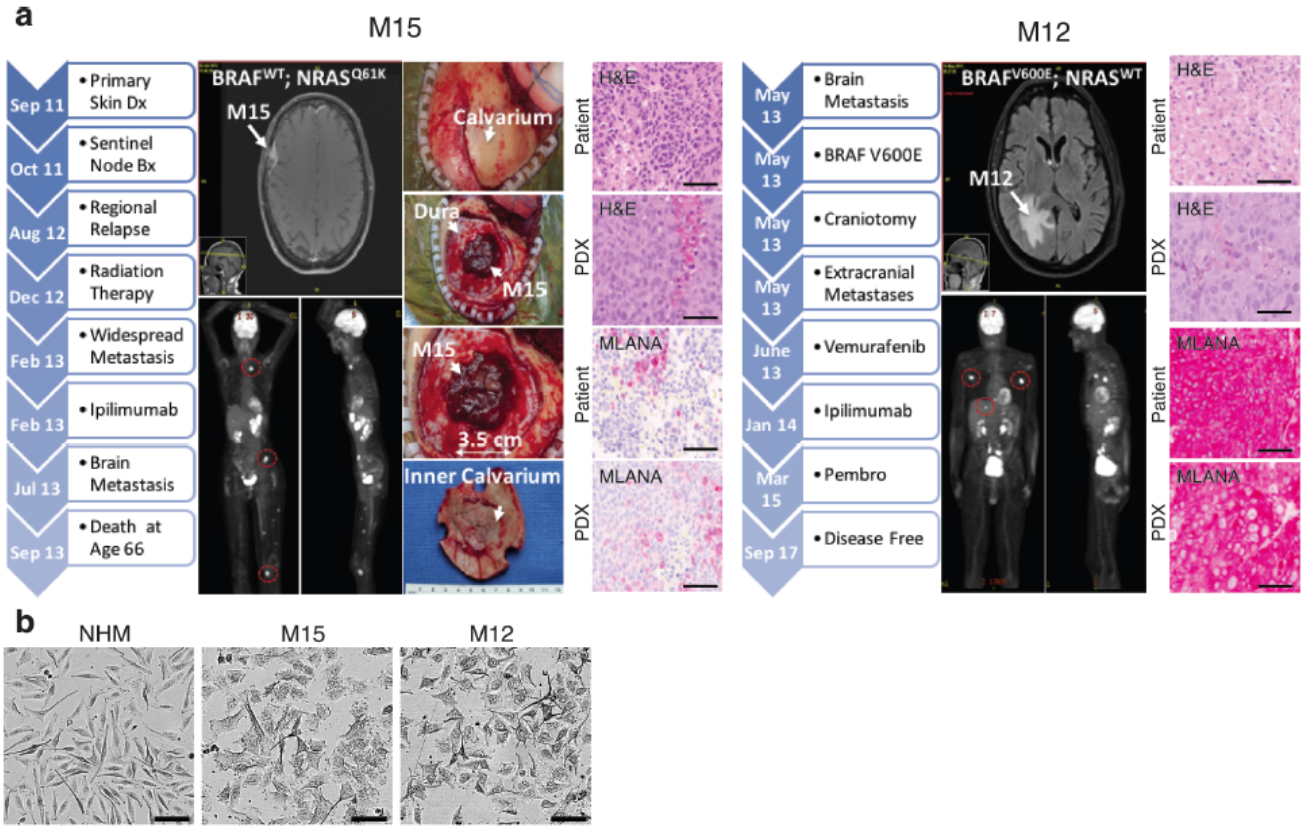

Supplementary Fig. 10. Characterization of patient-derived xenografts (PDX) used for single cell RNA-sequencing. To characterize gene expression at the single cell level, we made use of several established melanoma PDX (M12, M15 and M27). All PDX were derived from the brain metastases of Mayo Clinic patients. **a** Characterization of M15 and M12 patient-derived xenografts. The highly aggressive M15 melanoma metastasized widely and was unresponsive to immunotherapy whereas immunotherapy for M12 led to a complete remission that lasted for at least three years. Likewise, M27 had a complete and sustained response to therapy. Key characteristics of tumor histology were maintained in PDX, including expression levels of the MLANA (Melanoma Antigen Recognized by T-Cells 1) melanocyte lineage marker as demonstrated for the M12 and M15 PDX. **b** Representative phase contrast microscopy images of normal human melanocytes (NHM) and PDX M15 and M12 cultures. Both PDX lost their spindle cell shape compared to NHM. Scale bars 100 μm.

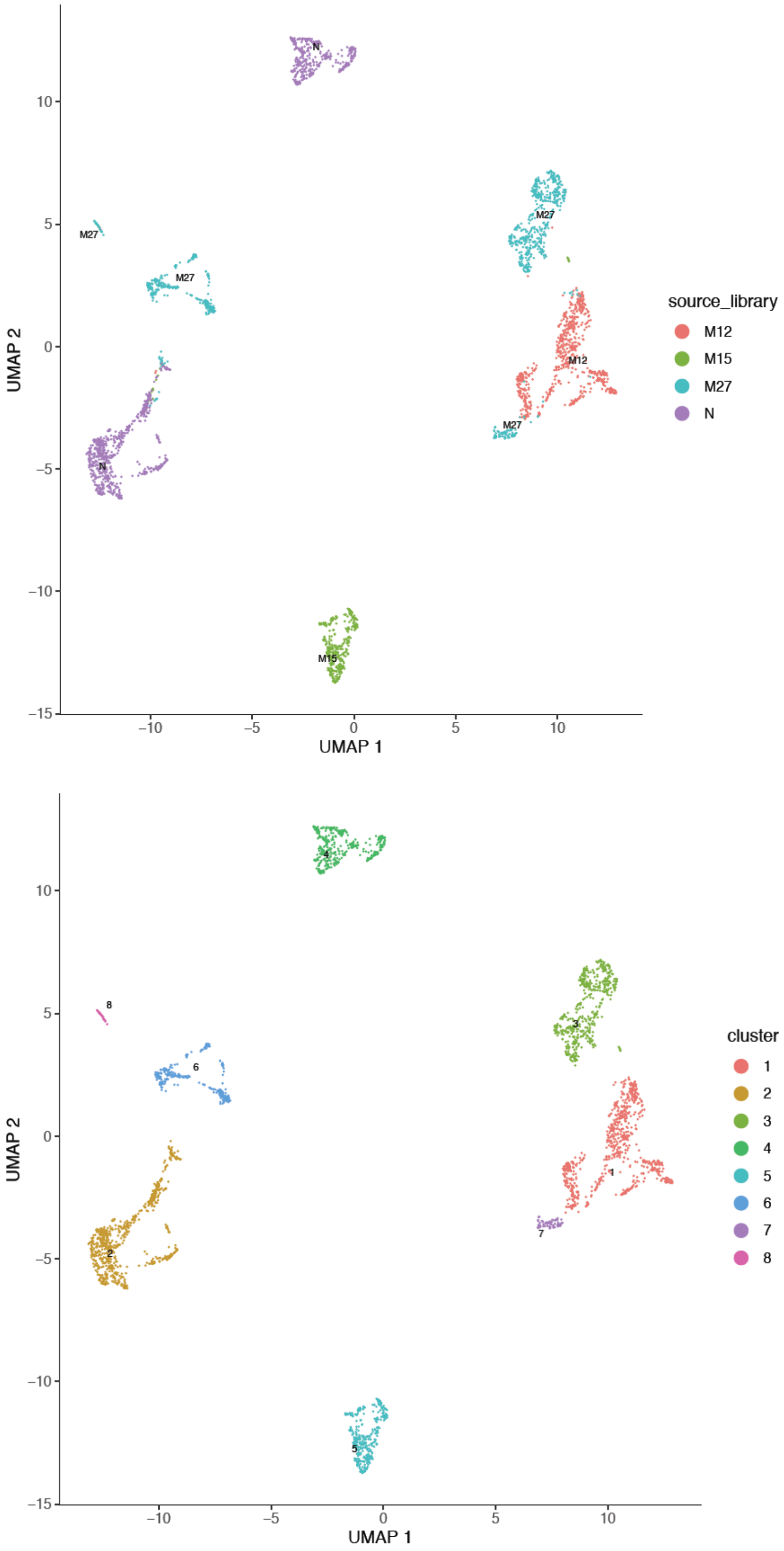

Supplementary Fig. 11. UMAP visualization of 3322 melanoma patient-derived xenograft cells and normal human neonatal epidermal melanocytes, colored by the sample they belong to (upper panel) and by cluster (lower panel).

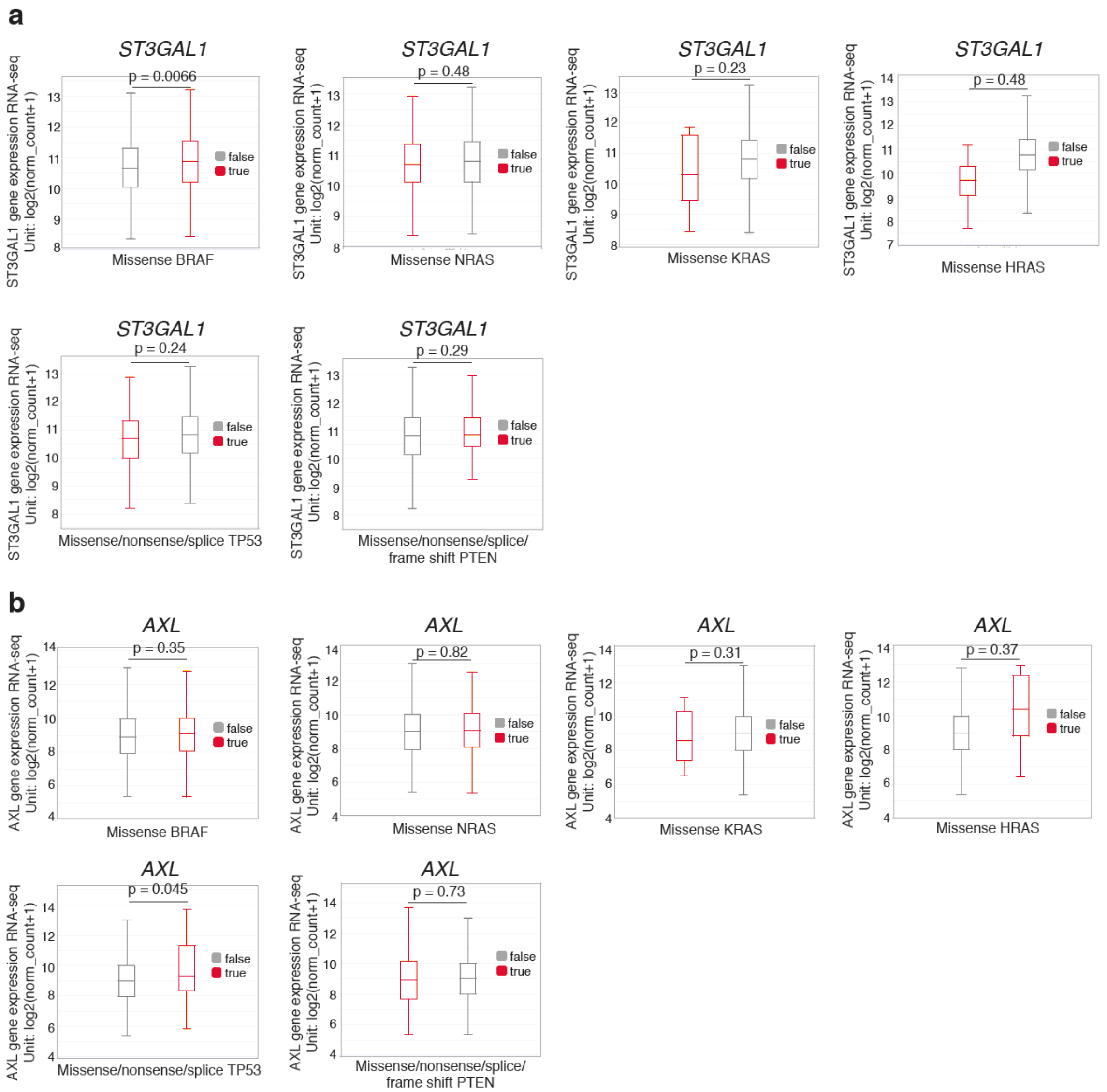

c

Mutated Driver	Significant Differential Expression	P-value
BRAF	Increased ST3GAL1	0.0066
NRAS	None	N/A
KRAS	None	N/A
HRAS	None	N/A
TP53	Increased AXL	0.045
PTEN	None	N/A

Supplementary Fig. 12. Correlations between *ST3GAL1* and *AXL* gene expression and mutational status of *BRAF, NRAS, KRAS, HRAS TP53* and *PTEN* in melanoma samples. a, b Box plots were created with Xena software using TCGA data comparing mRNA expression data for those cases with (red) or without (grey) genomic mutations in each gene. BRAF mutant group shows higher expression of *ST3GAL1* when compared to the BRAF (wild type) WT group. Likewise, TP53 mutant group shows higher expression of *AXL* when compared to the TP53 WT group. Box-plots report median (central lines), 25th and 75th percentiles (box limits), and upper and lower whiskers represent values no further than x1.5 interquartile range (IQR). Lines above and below the box show the minimum and maximum. The University of California Santa Cruz Xena platform was used to analyse correlation, transcriptomic and survival data from The Cancer Genome Atlas (TCGA) Melanoma (SKCM) cohort of 17 data sets (https://doi.org/10.1101/326470). Level 3 data was downloaded from TCGA data coordination center and gene-level transcription estimates were shown as log2(+1) transformed RSEM normalized count. Genes were mapped

onto the human genome coordinates using UCSC Xena HUGO probeMap. Available clinical data allowed for sorting of the SKCM dataset on the presence or absence of metastasis in the patient. Further analysis was carried out using only those cases positive for metastasis. **c** Summary of all significant changes in *ST3GAL1* and *AXL* mRNA expression in the presence or absence of various genomic mutations. *P* values were calculated by one-sided Welch's *t*-test (BRAF: n=239 mutant, n=242 WT; NRAS: n=126 mutant, n=355 WT; KRAS: n=11 mutant, n=470 WT; HRAS: n=5 mutant, n=476 WT; TP53: n=66 mutant, n=415 WT; PTEN: n=29 mutant, n= 452 WT).

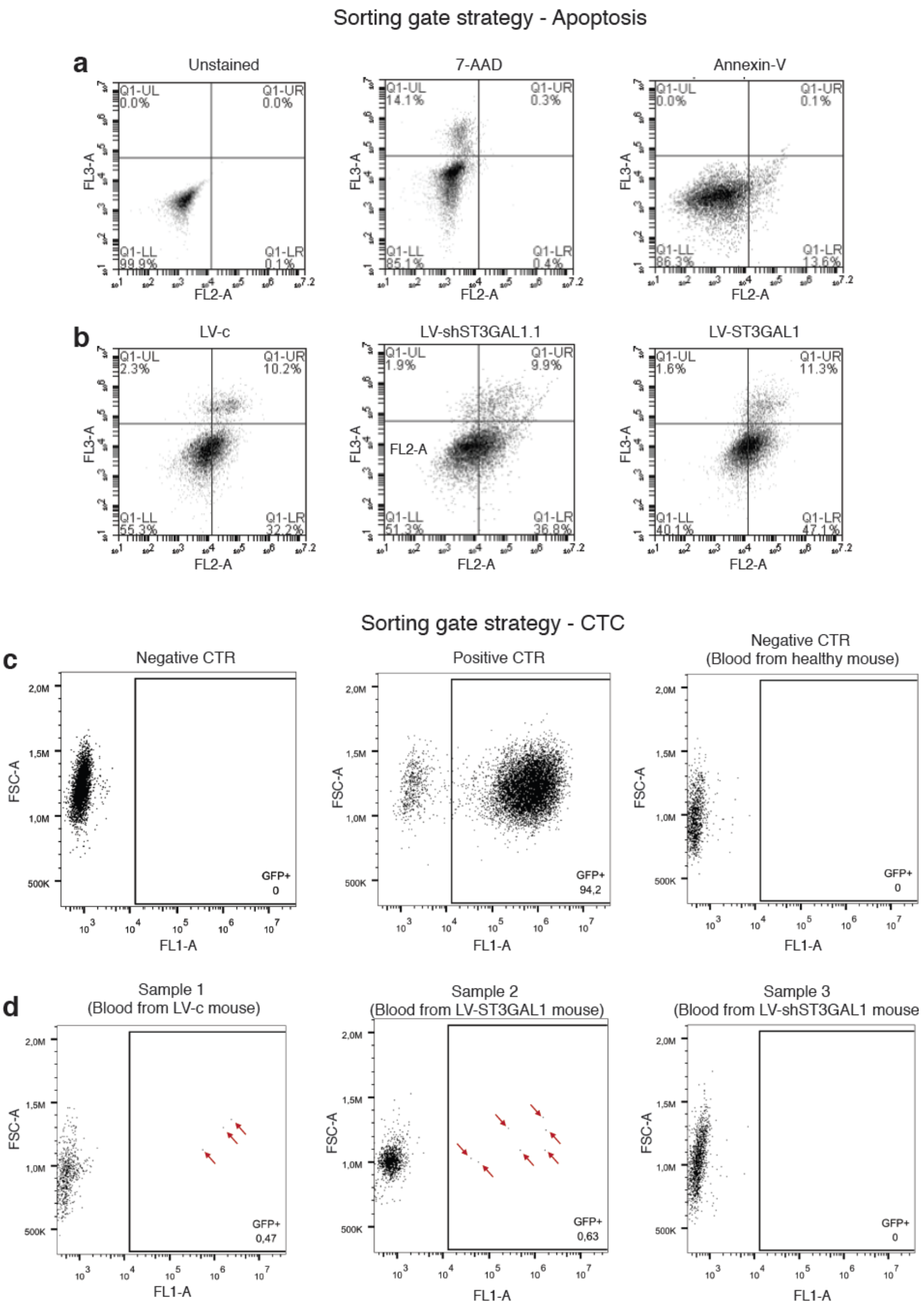

Supplementary Fig. 13. FACS sorting gate strategies. a, b FACS sorting strategy used for apoptosis analysis shown in Supplementary Figure 2b and e. Sorting gates were drawn using either unstained cells as negative control, Annexin-V$^+$ cells (FL2-A, PE-conjugated) and 7-AAD$^+$ cells (FL3-A, PC.5-conjugated) as the positive ones (**a**). Melanoma cells were then double-stained with Annexin-V and 7-AAD, and both early apoptotic fraction (Annexin-V$^+$ and 7-AAD$^-$, Q1-LR) and late (Annexin-V$^+$ and 7-AAD$^+$, Q1-UR) apoptotic fractions extracted. A representative experiment for SSM2c transduced with LV-c, LV-shST3GAL1.1 or LV-shST3GAL1.2 is shown in (**b**). **c, d** FACS sorting strategy used for the evaluation of CTC shown in Figure 5e. Sorting gates were drawn using A375 M6 GFP$^-$ and GFP$^+$ cells as negative or positive control, respectively (**c**). Blood sample from healthy mice was also used to exclude unspecific fluorescence signals from murine blood cells (**c**). Representative blood samples from mice injected with either LV-c, LV-ST3GAL1 or LV-shST3GAL1 are shown (**d**).

SUPPLEMENTARY TABLES

Supplementary Table 1. Human melanoma tissue microarrays and ST3GAL1 immunostaining.

A	B	C	D	E	F	G	H	I	J
Position	No.	Sex	Age	Organ	Pathology diagnosis	Stage	TNM	Type	ST3GAL1 expression
A1	1	M	71	Soft tissue	Malignant melanoma of right rump	II	T4N0M0	Malignant	+++
A2	2	M	60	Skin	Malignant melanoma of right rump	III	T4N1M0	Malignant	+
A3	3	F	16	Skin	Malignant melanoma of back	II	T4N0M0	Malignant	+
A4	4	M	61	Skin	Malignant melanoma of left oxter	IV	T4bN0M1	Malignant	+++
A5	5	M	55	Skin	Malignant melanoma of sole	II	T4N0M0	Malignant	-
A6	6	M	51	Skin	Malignant melanoma of back	II	T4N0M0	Malignant	++
A7	7	F	63	Skin	Malignant melanoma of right foot	II	T4N0M0	Malignant	+
A8	8	F	46	Skin	Malignant melanoma of thigh	II	T4N0M0	Malignant	-
A9	9	F	32	Skin	Malignant melanoma of right lumbar part	II	T4N0M0	Malignant	++
A10	10	M	80	Skin	Malignant melanoma of right sole	II	T4N0M0	Malignant	-
B1	11	F	42	Soft tissue	Malignant melanoma of right thigh	II	T4N0M0	Malignant	++
B2	12	M	50	Striated muscle	Malignant melanoma of left shoulder	II	T4N0M0	Malignant	+
B3	13	M	41	Skin	Malignant melanoma of left leg	II	T3N0M0	Malignant	+
B4	14	F	88	Skin	Malignant melanoma of left sole	II	T4N0M0	Malignant	++
B5	15	M	51	Skin	Malignant melanoma of chest wall	II	T4N0M0	Malignant	-
B6	16	M	52	Skin	Malignant melanoma of left lower quadrant	II	T4N0M0	Malignant	+
B7	17	F	42	Skin	Malignant melanoma of left thumb	II	T4N0M0	Malignant	++
B8	18	F	58	Skin	Malignant melanoma of left rump	II	T4N0M0	Malignant	++
B9	19	M	38	Skin	Malignant melanoma of right abdominal wall	III	T4N1M0	Malignant	++
B10	20	F	52	Skin	Malignant melanoma of left foot	II	T3N0M0	Malignant	+
C1	21	M	40	Skin	Malignant melanoma of right chest wall	II	T4N0M0	Malignant	+
C2	22	F	59	Skin	Malignant melanoma of anus	II	T4N0M0	Malignant	+
C3	23	F	77	Skin	Malignant melanoma of left sole	II	T4N0M0	Malignant	+++
C4	24	F	62	Skin	Malignant melanoma of right thumb	II	T4N0M0	Malignant	-
C5	25	F	66	Skin	Malignant melanoma of chest wall	II	T4N0M0	Malignant	-
C6	26	M	71	Skin	Malignant melanoma of right thigh	II	T2N0M0	Malignant	+++
C7	27	F	38	Skin	Malignant melanoma of right toe	II	T4N0M0	Malignant	+++
C8	28	F	83	Skin	Malignant melanoma of right little toe	II	T2N0M0	Malignant	++
C9	29	M	52	Skin	Malignant melanoma of right heel	IV	T4N1M1	Malignant	+
C10	30	M	51	Skin	Malignant melanoma of left rump	III	T4N1M0	Malignant	+++
D1	31	M	36	Skin	Malignant melanoma of back	II	T3N0M0	Malignant	-
D2	32	F	45	Soft tissue	Malignant melanoma of left thigh	II	T2N0M0	Malignant	+
D3	33	M	51	Skin	Malignant melanoma of left oxter	II	T4N0M0	Malignant	+++
D4	34	F	61	Skin	Malignant melanoma of left foot	II	T4N0M0	Malignant	+
D5	35	M	66	Skin	Malignant melanoma of left thigh	II	T4N0M0	Malignant	++
D6	36	M	55	Skin	Malignant melanoma of left upper arm	II	T4N0M0	Malignant	-
D7	37	M	65	Skin	Malignant melanoma of scalp	II	T4N0M0	Malignant	+++
D8	38	M	45	Skin	Malignant melanoma of anus	II	T4N0M0	Malignant	+
D9	39	F	73	Skin	Malignant melanoma of right big toe	II	T3N0M0	Malignant	++
D10	40	F	41	Vulva	Malignant melanoma of cunnus	II	T4N0M0	Malignant	+
E1	41	F	72	Vulva	Malignant melanoma of cunnus	I	T1N0M0	Malignant	+++
E2	42	F	72	Skin	Malignant melanoma of right cheek	II	T4N0M0	Malignant	+
E3	43	F	46	Skin	Malignant melanoma of right big toe	II	T4N0M0	Malignant	+
E4	44	F	47	Skin	Malignant melanoma of right upper arm	II	T4N0M0	Malignant	+++
E5	45	M	53	Skin	Malignant melanoma of right sole	II	T4N0M0	Malignant	+++
E6	46	F	56	Skin	Malignant melanoma of left leg	II	T3N0M0	Malignant	+
E7	47	F	74	Skin	Malignant melanoma of left heel	II	T2N0M0	Malignant	+
E8	48	M	65	Skin	Malignant melanoma of right thumb	II	T4N0M0	Malignant	++

E9	49	M	76	Skin	Malignant melanoma of right medial malleolus	III	T4N1M0	Malignant	++
E10	50	F	54	Skin	Malignant melanoma of left heel	II	T4N0M0	Malignant	+
F1	51	M	49	Skin	Malignant melanoma of left foot	III	T4N2M0	Malignant	-
F2	52	M	55	Skin	Malignant melanoma of right forearm	II	T4N0M0	Malignant	-
F3	53	M	74	Skin	Malignant melanoma of left sole	II	T4N0M0	Malignant	+++
F4	54	M	65	Skin	Malignant melanoma of right sole	II	T2N0M0	Malignant	+
F5	55	M	31	Skin	Malignant melanoma of scalp	II	T4N0M0	Malignant	++
F6	56	F	41	Skin	Malignant melanoma of scalp	II	T4N0M0	Malignant	++
F7	57	F	63	Lymph node	Metastatic malignant melanoma of right groin	-	-	Metastasis	+
F8	58	M	58	Skin	Metastatic malignant melanoma of left groin	-	-	Metastasis	-
F9	59	F	55	Skin	Metastatic malignant melanoma of left groin	-	-	Metastasis	++
F10	60	M	44	Lymph node	Metastatic malignant melanoma of right oxter	-	-	Metastasis	+++
G1	61	M	72	Skin	Metastatic malignant melanoma of right groin	-	-	Metastasis	-
G2	62	F	47	Skin	Metastatic malignant melanoma of armpit	-	-	Metastasis	++
G3	63	M	63	Adipose tissue	Metastatic malignant melanoma of right neck	-	-	Metastasis	+
G4	64	F	40	Lymph node	Metastatic malignant melanoma of right groin	-	-	Metastasis	++
G5	65	M	70	Lymph node	Metastatic malignant melanoma of armpit	-	-	Metastasis	+
G6	66	M	56	Soft tissue	Metastatic malignant melanoma of preauricula	-	-	Metastasis	++
G7	67	M	68	Lymph node	Metastatic malignant melanoma of neck	-	-	Metastasis	+++
G8	68	F	72	Soft tissue	Metastatic malignant melanoma of left groin	-	-	Metastasis	+
G9	69	F	41	Soft tissue	Metastatic malignant melanoma of groin	-	-	Metastasis	++
G10	70	F	61	Lymph node	Metastatic malignant melanoma of right groin	-	-	Metastasis	+++
H1	71	F	42	Lymph node	Metastatic malignant melanoma of groin	-	-	Metastasis	+
H2	72	F	43	Lymph node	Metastatic malignant melanoma of groin	-	-	Metastasis	-
H3	73	F	56	Lymph node	Metastatic malignant melanoma of groin	-	-	Metastasis	-
H4	74	F	56	Lymph node	Metastatic malignant melanoma of groin	-	-	Metastasis	+++
H5	75	F	49	Lymph node	Metastatic malignant melanoma of neck	-	-	Metastasis	+++
H6	76	F	41	Soft tissue	Metastatic malignant melanoma of right groin	-	-	Metastasis	++
H7	77	M	38	Skin	Intradermal nevus of right face	-	-	Benign	-
H8	78	F	29	Skin	Intradermal nevus of left shoulder	-	-	Benign	+
H9	79	F	50	Soft tissue	Intradermal nevus of back	-	-	Benign	+
H10	80	F	35	Skin	Intradermal nevus of chest wall	-	-	Benign	+
I1	81	F	23	Skin	Compound nevus of right waist	-	-	Benign	+
I2	82	M	25	Skin	Compound nevus of left leg	-	-	Benign	-
I3	83	M	20	Skin	Intradermal nevus of left shoulder	-	-	Benign	-
I4	84	M	19	Skin	Intradermal nevus of right cheek	-	-	Benign	-
I5	85	F	10	Skin	Compound nevus of right dorsum of foot	-	-	Benign	+
I6	86	F	2	Skin	Intradermal nevus of frontal region	-	-	Benign	+
I7	87	M	6	Skin	Compound nevus of left face	-	-	Benign	-
I8	88	M	5 m.	Skin	Intradermal nevus of face	-	-	Benign	-
I9	89	M	25	Skin	Intradermal nevus of scalp	-	-	Benign	+
I10	90	M	46	Skin	Intradermal nevus of back	-	-	Benign	+
J1	91	F	2	Skin	Intradermal nevus of left leg	-	-	Benign	-
J2	92	M	42	Skin	Intradermal nevus of face	-	-	Benign	+
J3	93	M	11	Skin	Pigmented nevus of left thigh	-	-	Benign	ND
J4	94	F	30	Skin	Compound nevus of right arm	-	-	Benign	+
J5	95	M	62	Skin	Junctional nevus of abdominal wall	-	-	Benign	-
J6	96	M	39	Skin	Compound nevus of scalp	-	-	Benign	+
J7	97	F	7	Skin	Junctional nevus of right forearm	-	-	Benign	+

J8	98	M	50	Skin	Compound nevus of upper arm	-	-	Benign	+
J9	99	M	53	Skin	Sebaceous nevus of face	-	-	Benign	-
J10	100	M	32	Skin	Sebaceous nevus of right elbow	-	-	Benign	-
		F	47	Lymph node	Metastatic malignant melanoma of armpit	.	.	Metastasis	++
		M	58	Lymph node	Metastatic malignant melanoma of left groin	-	-	Metastasis	-
		M	54	Lymph node	Metastatic malignant melanoma of left groin	-	-	Metastasis	+++
		F	72	Lymph node	Metastatic malignant melanoma of left groin	-	-	Metastasis	++
		F	41	Lymph node	Metastatic malignant melanoma of right groin	-	-	Metastasis	++
		M	56	Lymph node	Metastatic malignant melanoma of left preauricula	-	-	Metastasis	+++
		M	49	Lymph node	Metastatic malignant melanoma of pelvic cavity	-	-	Metastasis	++
		M	63	Lymph node	Metastatic malignant melanoma of right neck	-	-	Metastasis	+++
		F	41	Lymph node	Metastatic malignant melanoma of groin	-	-	Metastasis	++
		M	46	Lymph node	Metastatic malignant melanoma of right groin	-	-	Metastasis	++
		F	40	Lymph node	Metastatic malignant melanoma of right groin	-	-	Metastasis	+++
		M	70	Lymph node	Metastatic malignant melanoma of armpit	-	-	Metastasis	+++
		F	61	Lymph node	Metastatic malignant melanoma of right groin	-	-	Metastasis	+
		F	43	Lymph node	Metastatic malignant melanoma of groin	-	-	Metastasis	++
		F	56	Lymph node	Metastatic malignant melanoma of groin	-	-	Metastasis	+++
		M	68	Lymph node	Metastatic malignant melanoma of neck	-	-	Metastasis	+
		F	49	Lymph node	Metastatic malignant melanoma of neck	-	-	Metastasis	+
		F	38	Lymph node	Metastatic malignant melanoma of right groin	-	-	Metastasis	++
		M	73	Lymph node	Metastatic malignant melanoma of right groin	-	-	Metastasis	+++
		M	51	Lymph node	Metastatic malignant melanoma of left upper arm	-	-	Metastasis	+++

a) position of core in the array; **b**) core number; **c**) gender; **d**) age; **e**) location of the tumor; **f**) pathology diagnosis; **g, h**) stage and TNM grading according to AJCC-TNM classification (http://www.biomax.us/tissue-arrays/Melanoma/ME1004a); **i**) type of tumor; **j**) immunohistochemical ST3GAL1 expression based on intensity (arbitrary units: -, negative staining; +, low staining; ++, medium staining; +++, high staining). ND: non-evaluable core due to ruined tissue or extensive necrosis.

Supplementary Table 2. List of ST3GAL1 sialylated targets.

Accession Number	Protein Description	LV-shST3GAL1 Average LFQ Intensity (arbitrary units)	LV-ST3GAL1 Average LFQ Intensity (arbitrary units)	Fold Change	P-value
P30530	Tyrosine-protein kinase receptor AXL (UFO)	4.28E+07	6.20E+07	1.5	3.18E-03
P26232	Catenin alpha-2	3.10E+07	4.74E+07	1.5	4.56E-04
P35221	Catenin alpha-1	1.05E+08	1.55E+08	1.5	6.49E-04
P35222	Catenin beta-1	1.31E+08	1.94E+08	1.5	5.38E-05
Q9Y653	Adhesion G-protein coupled receptor G1 (ADGRG1)	5.89E+06	1.26E+07	2.1	7.17E-04
P00533	Epidermal growth factor receptor (EGFR)	1.37E+08	2.09E+08	1.5	9.80E-06
P08138	Nerve growth factor receptor (NGFR)	4.29E+06	1.63E+07	3.8	8.78E-05
P23229	Integrin alpha-6	2.58E+08	4.00E+08	1.6	1.21E-05
P16144	Integrin beta-4	4.47E+08	6.91E+08	1.6	6.19E-06
Q92896	Golgi apparatus protein 1 (GLG1)	8.90E+07	1.98E+08	2.2	4.22E-05
P07686	Beta-hexosaminidase subunit beta (HEXB)	1.80E+07	2.66E+07	1.5	1.72E-04
P10586	Receptor-type tyrosine-protein phosphatase F (PTPRF)	5.88E+07	1.05E+08	1.8	4.63E-05
P13726	Tissue factor 3 (TF3)	4.82E+06	8.06E+06	1.7	4.99E-03

Proteins identified as MAL-bound that are involved in biological processes relevant to metastasis, such as cell motility, cell migration and locomotion control mechanisms (see Fig. 7b). The parameter "LFQ" was calculated by the software and was used to estimate the abundance of the proteins across the different samples. Two-tailed unpaired Student's *t*-test was used to highlight proteins with a significant ($p \leq 0.05$) different abundance in LV-shST3GAL1 and LV-ST3GAL1 samples. A fold change ≥ 1.5 was selected to classify proteins as potential substrates of ST3GAL1.

Supplementary Table 3. List of melanoma cell lines used in this study and their genetic alterations.

Cell line	Mutations	Characteristics
A2058	BRAF V600E	Metastatic melanoma (skin)
A375	BRAF V600E	Metastatic melanoma (skin)
A375 M6	BRAF V600E	Obtained from a lung metastasis of A375 cells
SK-Mel-2	NRAS Q61R	Metastatic melanoma (skin)
SK-Mel-5	BRAF V600E	Metastatic melanoma (lymph node)
SK-Mel-28	BRAF V600E	Metastatic melanoma (skin)
SK-Mel-197	wt BRAF	Metastatic melanoma (skin)
501-Mel	BRAF V600E	Metastatic melanoma
MeWo	NF1, p53, CDKN2A trunc/del	Metastatic melanoma (lymph node)
SSM2c	wt BRAF / wt NRAS	Primary cell line, obtained from a metastatic melanoma
M51	wt BRAF / wt NRAS	Primary cell line, obtained from a metastatic melanoma

Supplementary Table 4. List of primers used in this study.

Primer	Sequence (5’ to 3’)
ST3GAL1-F	CTTCCGGGAGCTGGGAGATA
ST3GAL1-R	TCTTTGCAGGAACCGGGATG
GALNT3-F	AGCGTTGGTCAGCCTCTATG
GALNT3-R	TCAAAGTACTGGTTTCCCCCAA
GALNT6-F	CTTTTGAGTTCGCCAAGCCC
GALNT6-R	AGCAAACGTCGGGGATTTGA
GALNT12-F	GCGGCTGCACCAGATTAACAT
GALNT12-R	CTCTTTGCACAGCGGGTTCC
GCNT1-F	GTTTATGCATCGTGGAGCCG
GCNT1-R	GGCATCCTCTCCGTTTCCAG
GCNT2-F	AGAACAACAGCGTTGAAACCG
GCNT2-R	AGCTCTGAGGTTTCCAGTCC
GLI1-F	CCCAGTACATGCTGGTGGTT
GLI1-R	GCTTTACTGCAGCCCTCGT
HPRT-F	GCCAGACTTTGTTGGATTTG
HPRT-R	CTCTCATCTTAGGCTTTGTATTTTG
SOX2-F	GAGCTTTGCAGGAAGTTTGC
SOX2-R	GCAAGAAGCCTCTCCTTGAA
TBP-F	CAACAGCCTGCCACCTTAC
TBP-R	CTGAATAGGCTGTGGGGTC
ST3GAL1enhI-F	ATCCCAAAGTCCCAGTGCTC
ST3GAL1enhI-R	GGAACCGACCTCCCACTAGA
ST3GAL1enhII-F	GTCCATCCTGCCCACCCTAA
ST3GAL1enhII-R	TTGGCTTTCTTCATGGGCAC
ST3GAL1prom-F	CGCAACTTTTCGGAGGAATGT
ST3GAL1prom-R	CGCTGGAAGGATGCAATGAC
ACTINprom-F	TCGAGCCATAAAAGGCAACT
ACTINprom-R	CTTCCTCAATCTCGCTCTCG

Supplementary Table 5. List of antibodies used in this study.

Antibody (use)	Source	Cat. No.	Dilution
ST3GAL1 (WB)	R&D System	AF6905	1:2000
ST3GAL1 (IHC)	Invitrogen	PA5-21721	1:50
GFP (IHC)	Santa Cruz Biotechnology	sc-9996	1:500
SOX2 (WB)	Santa Cruz Biotechnology	sc-365964	1:1000
AXL (WB)	Santa Cruz Biotechnology	sc-166269	1:1000
GLI1 (WB)	Cell Signaling Technology	#2643	1:1000
phospho-AXL Y702 (WB)	Cell Signaling Technology	#5724	1:1000
Beta-catenin (WB)	Cell Signaling Technology	#8480	1:1000
integrin α5 (WB)	Cell Signaling Technology	#4705	1:1000
integrin β4 (WB)	Cell Signaling Technology	#14803	1:1000
N-cadherin (WB)	Cell Signaling Technology	#13116	1:1000
phospho-Tyr 100 (WB)	Cell Signaling Technology	#9411	1:1000
EGFR (WB)	Cell Signaling Technology	#8504	1:1000
NGFR (WB)	Cell Signaling Technology	#4201	1:1000
SNAIL (WB)	Cell Signaling Technology	#3879	1:1000
SLUG (WB)	Cell Signaling Technology	#9585	1:1000
Vimentin (WB)	Cell Signaling Technology	#5741	1:1000
BCL-2 (WB)	Cell Signaling Technology	#15071	1:1000
BAX (WB)	Cell Signaling Technology	#2772	1:1000
PARP-1 (WB)	Cell Signaling Technology	#9542	1:1000
ACTIN (WB)	Santa Cruz Biotechnology	sc-69879	1:5000
GAPDH (WB)	Santa Cruz Biotechnology	sc-20357	1:5000
HSP90 (WB)	Santa Cruz Biotechnology	sc-13119	1:10000
anti-mouse (WB)	Cell Signaling Technology	#7076	1:5000
anti-rabbit (WB)	Cell Signaling Technology	#7074	1:5000
anti-goat (WB)	R&D System	HAF109	1:4000
anti-sheep (WB)	R&D System	HAF016	1:4000

WB: Western blotting; IHC: Immunohistochemistry.
