# Supplementary material for: ST3GAL1 is a target of the SOX2-GLI1 transcriptional complex and promotes melanoma metastasis through AXL
Source: Nat Commun. 2020 Nov 17;11:5865. doi: 10.1038/s41467-020-19575-2 (PMC7673140; doi:10.1038/s41467-020-19575-2)
Supplement: Supplementary file 3 — Description of Additional Supplementary Files [file 41467_2020_19575_MOESM3_ESM.docx]

**Description of Additional Supplementary Files**

**Supplementary Data 1**

RNA-sequencing data of SOX2- and GLI1-depleted cells. Results from RNA-sequencing were filtered as follows: upregulated (Log2FC>0.58), downregulated (Log2FC<-0.58), and unchanged (0.58>Log2FC>-0.58). Differentially regulated refers to a list of either up or downregulated genes. Sheet 1 (Data S1a): list of 751 commonly down-regulated genes in LV-shGLI1 and LV-shSOX2 cells (FDR<0.1). Sheet 2 (Data S1b): list of 230 commonly up-regulated genes in LV-shGLI1 and LV-shSOX2 cells (FDR<0.1). Sheet 3 (Data S1c): list of 1682 genes differentially regulated in LV-shGLI1 cells and unchanged in LV-shSOX2 cells (FDR<0.1). Sheet 4 (Data S1d): List of 2302 of genes differentially regulated in LV-shSOX2 cells and unchanged in LV-shGLI1 cells (FDR<0.1).

**Supplementary Data 2**

Enrichment of MAL-bound proteins in A375 M6 melanoma cells expressing ST3GAL1 identified by mass spectrometry. Sheet 1 (Proteins significantly enriched): table lists all proteins significantly enriched (Fold Change ≥ 1.5 and p-value ≤ 0.05) by the MAL lectin affinity chromatography experiment. Sheet 2 (Original data): table lists al proteins identified in the proteomics experiment. Protein ID, number of proteins per group, number of peptides, razor peptides and unique peptides are reported together with sequence coverage, sequence lengths, Q-values, average LFQ intensities and MS/MS counts.

**Supplementary Data 3**

Single cell RNA-sequencing data of PDXs and normal human epidermal melanocytes were analyzed by R package Monocle3. There are four libraries: normal human epidermal melanocytes (labeled N) and in 3 patient-derived xenografts (PDX) (labeled M12, M15 and M27). Cluster markers for each cluster are determined by comparing the gene expression in the cluster of interest against all other clusters combined. Library markers are determined in a similar way by comparing each library against the rest combined.

Sheet 1: Normalized expression of genes *AXL, GLI1, ST3GAL1* and *SOX2*, averaged by library.

Sheet 2: Normalized expression of genes *AXL, GLI1, ST3GAL1* and *SOX2*, averaged by cluster.

Sheet 3: Normalized expression of Cluster 1 marker genes, averaged by cluster.

Sheet 4: Normalized expression of Cluster 2 marker genes, averaged by cluster.

Sheet 5: Normalized expression of Cluster 3 marker genes, averaged by cluster.

Sheet 6: Normalized expression of Cluster 4 marker genes, averaged by cluster.

Sheet 7: Normalized expression of Cluster 5 marker genes, averaged by cluster.

Sheet 8: Normalized expression of Cluster 6 marker genes, averaged by cluster.

Sheet 9: Normalized expression of Cluster 7 marker genes, averaged by cluster.

Sheet 10: Normalized expression of Cluster 8 marker genes, averaged by cluster.

Sheet 11: Normalized expression of library N marker genes, averaged by library.

Sheet 12: Normalized expression of library M15 marker genes, averaged by library.

Sheet 13: Normalized expression of library M12 marker genes, averaged by library.

Sheet 14: Normalized expression of library M27 marker genes, averaged by library.
