# Supplementary material for: ST3GAL1 is a target of the SOX2-GLI1 transcriptional complex and promotes melanoma metastasis through AXL
Source: Nat Commun. 2020 Nov 17;11:5865. doi: 10.1038/s41467-020-19575-2 (PMC7673140; doi:10.1038/s41467-020-19575-2)
Supplement: Supplementary file 7 — Reporting Summary [file 41467_2020_19575_MOESM7_ESM.pdf]

nature research

Corresponding author(s): Barbara Stecca
Last updated by author(s): Oct 15, 2020

Reporting Summary

Nature Research wishes to improve the reproducibility of the work that we publish. This form provides structure for consistency and transparency in reporting. For further information on Nature Research policies, see our Editorial Policies and the Editorial Policy Checklist.

Statistics

For all statistical analyses, confirm that the following items are present in the figure legend, table legend, main text, or Methods section.

n/a	Confirmed	
☐	☒	The exact sample size (n) for each experimental group/condition, given as a discrete number and unit of measurement
☐	☒	A statement on whether measurements were taken from distinct samples or whether the same sample was measured repeatedly
☐	☒	The statistical test(s) used AND whether they are one- or two-sided<br>*Only common tests should be described solely by name; describe more complex techniques in the Methods section.*
☒	☐	A description of all covariates tested
☐	☒	A description of any assumptions or corrections, such as tests of normality and adjustment for multiple comparisons
☐	☒	A full description of the statistical parameters including central tendency (e.g. means) or other basic estimates (e.g. regression coefficient) AND variation (e.g. standard deviation) or associated estimates of uncertainty (e.g. confidence intervals)
☒	☐	For null hypothesis testing, the test statistic (e.g. F, t, r) with confidence intervals, effect sizes, degrees of freedom and P value noted<br>*Give P values as exact values whenever suitable.*
☒	☐	For Bayesian analysis, information on the choice of priors and Markov chain Monte Carlo settings
☒	☐	For hierarchical and complex designs, identification of the appropriate level for tests and full reporting of outcomes
☒	☐	Estimates of effect sizes (e.g. Cohen's d, Pearson's r), indicating how they were calculated

Our web collection on statistics for biologists contains articles on many of the points above.

Software and code

Policy information about availability of computer code

Data collection	CytExpert Software (Beckman Coulter) version 2.3 (2011-2018)<br>M3Vision software (Biospace Lab) version 2.11.0<br>TFBIND bioinformatics software (http://tfbind.hgc.jp) version 1
Data analysis	FlowJo software (TreeStar Inc.) version 10.6.2<br>ImageJ version 1.51s<br>Microsoft Excel version 2016<br>GraphPad Prism version 6 and 7<br>MaxQuant software version 1.5.1.2.<br>Imaging software NIS Elements (Nikon) version 4.60<br>HCS collection software version 3.3.52<br>UCSC Xena HUGO probeMap version 13.3<br>Monocle3 version 0.2.2<br>UMAP algorithm (https://arxiv.org/abs/1802.03426) version 2

For manuscripts utilizing custom algorithms or software that are central to the research but not yet described in published literature, software must be made available to editors and reviewers. We strongly encourage code deposition in a community repository (e.g. GitHub). See the Nature Research guidelines for submitting code & software for further information.

Data

Policy information about availability of data

All manuscripts must include a data availability statement. This statement should provide the following information, where applicable:

- Accession codes, unique identifiers, or web links for publicly available datasets
- A list of figures that have associated raw data
- A description of any restrictions on data availability

RNAseq primary data that support the findings of this study have been deposited to GEO (accession number GSE159049; https://www.ncbi.nlm.nih.gov/geo/query/acc.cgi?acc=GSE159049). Processed RNAseq data are provided as Supplementary Data 1 and 3.
Expression data were obtained from public available microarrays from GSE46517 (https://www.ncbi.nlm.nih.gov/geo/query/acc.cgi?acc=GSE46517), GSE7553 (https://www.ncbi.nlm.nih.gov/geo/query/acc.cgi?acc=GSE7553), GDS1375 (https://www.ncbi.nlm.nih.gov/sites/GDSbrowser?acc=GDS1375) and GDS3966 (https://www.ncbi.nlm.nih.gov/sites/GDSbrowser?acc=GDS3966). Transcriptomic and genetic data were obtained from The Cancer Genome Atlas (TCGA) Melanoma (SKCM) cohort of 17 data sets (http://cancergenome.nih.gov/).
The source data underlying Figs. 1a, e-h; 2e, f; 3a-d, f-i; 4b, c, e, f, h; 5b-e, g, h; 6b, c, e-j; 7c, e-h; 8a-c, h and Supplementary Figs. 2a-f; 5a-j; 7a-c, e, f; 8a-d, are provided as Source Data file. All the other data supporting the findings of this study are available within the article, the Supplementary Information file and the Source Data file. A Reporting Summary for this article is available as Supplementary Information file.

Field-specific reporting

Please select the one below that is the best fit for your research. If you are not sure, read the appropriate sections before making your selection.

- [x] Life sciences
- [ ] Behavioural & social sciences
- [ ] Ecological, evolutionary & environmental sciences

For a reference copy of the document with all sections, see nature.com/documents/nr-reporting-summary-flat.pdf

Life sciences study design

All studies must disclose on these points even when the disclosure is negative.

Sample size	In vivo sample sizes were chosen based on prior studies, which validated the minimum number of mice to determine a significant difference. This was determined at a minimum of 8 mice for each group.<br>All in vitro sample sizes were performed in triplicate and independently for a minimum of 3 times. All attempts at replication were successful and provided an overall reflection of each individual experiment's findings.
Data exclusions	No data were excluded from the analysis.
Replication	Three technical replicates or more were performed for each experiments and experiments were repeated at least three times with similar results. All attempts at replication were successful.<br>H&E staining and GFP immunohistochemistry in lung metastases were repeated independently in serial sections at least three times and showed similar results.
Randomization	No experimental grouping requiring randomization was performed.
Blinding	RNA-seq, scRNA-seq, mass spectrometry and all animal procedures were carried out as blinded experiments to the group allocation during data collection and/or analysis. No blinding was performed for in vitro experiments and immunohistochemistry, as macroscopic and/or microscopic phenotypes prevented blinding during data collection and/or analysis. Standard protocols were equally applied on all samples.

Reporting for specific materials, systems and methods

We require information from authors about some types of materials, experimental systems and methods used in many studies. Here, indicate whether each material, system or method listed is relevant to your study. If you are not sure if a list item applies to your research, read the appropriate section before selecting a response.

Materials & experimental systems

n/a	Involved in the study	
[ ]	[x]	Antibodies
[ ]	[x]	Eukaryotic cell lines
[x]	[ ]	Palaeontology and archaeology
[ ]	[x]	Animals and other organisms
[ ]	[x]	Human research participants
[x]	[ ]	Clinical data
[x]	[ ]	Dual use research of concern

Methods

n/a	Involved in the study	
[x]	[ ]	ChIP-seq
[ ]	[x]	Flow cytometry
[x]	[ ]	MRI-based neuroimaging

Antibodies

Antibodies used

Western blotting:
Sheep anti human ST3GAL1 (1:2000; R&D System AF6905)
Mouse anti human, mouse, rat SOX2 (1:1000; Santa Cruz Biotechnology sc-365964)
Mouse anti human AXL (1:1000; Santa Cruz Biotechnology sc-166269)
Mouse anti human GLI1 (1:1000; Cell Signaling Technology #2643)
Rabbit anti human phospho-AXL Y702 (1:1000; Cell Signaling Technology #5724)
Rabbit anti human, mouse, rat, monkey β-catenin (1:1000; Cell Signaling Technology #8480)
Rabbit anti human, mouse, monkey Integrin α5 (1:1000; Cell Signaling Technology #4705)
Rabbit anti human Integrin β4 (1:1000; Cell Signaling Technology #14803)
Rabbit anti human, mouse N-cadherin (1:1000; Cell Signaling Technology #13116)
Mouse anti all species expected phospho-Tyr 100 (1:1000; Cell Signaling Technology #9411)
Rabbit anti human, mouse, monkey EGFR (1:1000; Cell Signaling Technology #8504)
Rabbit anti human, mouse, rat NGFR (1:1000; Cell Signaling Technology #4201)
Rabbit anti human, mouse, rat, monkey SNAIL (1:1000; Cell Signaling Technology #3879)
Rabbit anti human, mouse SLUG (1:1000; Cell Signaling Technology #9585)
Rabbit anti human, mouse, rat, monkey Vimentin (1:1000; Cell Signaling Technology #5741)
Mouse anti human BCL-2 (1:1000; Cell Signaling Technology #15071)
Rabbit anti human, mouse, rat, monkey BAX (1:1000; Cell Signaling Technology #2772)
Rabbit anti human, mouse, rat, monkey PARP-1 (1:1000; Cell Signaling Technology #9542)
Mouse anti human β-Actin (1:5000; Santa Cruz Biotechnology sc-69879)
Goat anti human GAPDH (1:5000; Santa Cruz Biotechnology sc-20357)
Mouse anti human, mouse, rat HSP90 (1:10000; Santa Cruz Biotechnology sc-13119)
Horse anti mouse IgG (1:5000; Cell Signaling Technology #7076)
Goat anti rabbit IgG (1:5000; Cell Signaling Technology #7074)
Donkey anti goat IgG (1:4000; R&D System HAF109)
Donkey anti sheep IgG (1:4000; R&D System HAF016)

Immunohistochemistry:
Rabbit Anti human ST3GAL1 (1:50; Invitrogen PA5-21721)
Mouse Anti GFP (1:500; Santa Cruz Biotechnology sc-9996)

Chromatin immunoprecipitation:
anti-SOX2 (1:000; R&D System, #MAB2018)
anti-GLI1 (1:500; Cell Signaling, #2643)
normal mouse IgG (1:100; Santa Cruz Biotechnology, #sc-2025).

Validation

Western blotting:
Sheep anti human ST3GAL1 – Validated by the manufacturer using human placenta lysate and by our group in ST3GAL1-silenced/overexpressing cells.
Mouse anti human, mouse, rat SOX2 – Validated by the manufacturer using F9 and H69AR whole cell lysates and by our group in SOX2-silenced/overexpressing cells.
Mouse anti human AXL – Validated by the manufacturer in Caki-1, FHs 173We, HUV-EC-C and A375 whole cell lysates and by our group in AXL-silenced/overexpressing cells.
Mouse anti human GLI1 – Validated by the manufacturer in RMS-13 and TOV-112D cell lysates and by our group in GLI1-silenced/overexpressing cells.
Rabbit anti human phospho-AXL Y702 – Validated by the manufacturer in NCI-H1299 cell lysate.
Rabbit anti human, mouse, rat, monkey β-catenin – Validated by the manufacturer in HeLa, 293T, NIH3T3 and C6 cell lysates.
Rabbit anti human, mouse, monkey Integrin α5 – Validated by the manufacturer in A549, NIH3T3 and COS cell lysates.
Rabbit anti human Integrin β4 – Validated by the manufacturer in HT-29, LNCaP and A-204 cell lysates.
Rabbit anti human, mouse N-cadherin – Validated by the manufacturer in A172 and MCF7 cell lysates.
Mouse anti all species expected phospho-Tyr 100 – Validated by the manufacturer in Jurkat cell lysate.
Rabbit anti human, mouse, monkey EGFR – Validated by the manufacturer in HeLa and A-431 cell lysates.
Rabbit anti human, mouse, rat NGFR - Validated by the manufacturer in RN33B and SW480 cell lysates and by our group in NGFR-silenced/overexpressing cells.
Rabbit anti human, mouse, rat, monkey SNAIL - Validated by the manufacturer in HCT116, HeLa, NIH3T3, Rat2 and COS cell lysates.
Rabbit anti human, mouse SLUG - Validated by the manufacturer in A204, SKMel 5 and NIH3T3 cell lysates.
Rabbit anti human, mouse, rat, monkey Vimentin - Validated by the manufacturer in HeLa, NIH3T3, C6 and COS-7 cell lysates.
Mouse anti human BCL-2 - Validated by the manufacturer in HeLa, RL-7, RPMI 8226, Jurkat, U266, HDLM-2, KARPAS-299, L-540, HT-29 cell lysates.
Rabbit anti human, mouse, rat, monkey BAX - Validated by the manufacturer in HeLa, 293, COS, L929, PC12 cell lysates.
Rabbit anti human, mouse, rat, monkey PARP-1 - Validated by the manufacturer in Jurkat and NIH3T3 cell lysates.
Mouse anti human β-Actin - Validated by the manufacturer in A-10, 293T, C32, HeLa and A-431 cell lysates.
Goat anti human GAPDH - Validated by the manufacturer in U-87 MG and SK-BR-3 cell lysates
Mouse anti human, mouse, rat HSP90 - Validated by the manufacturer in A-431, Hep G2, Jurkat, SK-BR-3 and K-562 cell lysates.

Immunohistochemistry:
Rabbit anti human ST3GAL1 – Validated by the manufacturer in paraffin-embedded human ovarian cancer tissue and by our group in ST3GAL1 overexpressing cells.
Mouse anti GFP - Validated by manufacturer and by our group in paraffin-embedded murine lung sections overexpressing GFP.

Chromatin immunoprecipitation:
anti-SOX2 – Validated by the manufacturer and by the group using SOX2-silenced melanoma cells.
anti-GLI1 – Validated by the manufacturer and by the group using GLI1-silenced melanoma cells.
Normal mouse IgG – Validated by the manufacturer.

Eukaryotic cell lines

Policy information about cell lines

Cell line source(s)	Commercial A375, SK-Mel-2, SK-Mel-5, SK-Mel-28, MeWo and HEK-293T cells were obtained from ATCC. 501-Mel, A2058 and SK-Mel-197 were kindly provided by Dr Laura Poliseno (CNR, Pisa, Italy). A375 M6 cells were isolated from lung metastases after tail-vein injection of A375 cells in SCID bg/bg mice (DOI: 10.1007/s00109-017-1590-9). Patient-derived melanoma cells SSM2c and M51 were >100 passages and were established from metastatic melanomas (SSM2c: DOI 10.1002/stem.1160; M51: DOI 10.1038/onc.2016.481). Short-term (passage 1-2) patient-derived melanoma cells Me-14, Me-16, Me-17, Me-25, Me-28, Me-32, Me-41, Me-42 and Me-44 were established from primary or metastatic melanomas.<br>Short-term cultures of M12, M15 and M27 for scRNA-seq were generated from PDXs tumors and derived from melanoma brain metastasis.
Authentication	Cell lines were authenticated by short tandem repeat (STR) analysis.
Mycoplasma contamination	Mycoplasma contamination was regularly verified by PCR. All cells were find to be negative.
Commonly misidentified lines<br>(See ICLAC register)	No commonly misidentified cell lines were used in this study.

Animals and other organisms

Policy information about studies involving animals; ARRIVE guidelines recommended for reporting animal research

Laboratory animals	Female athymic nude mice 6-8 week old (Charles River Italy).<br>Mice were maintained at the animal facility (CeSAL, Centro Stabulazione Animali da Laboratorio) of the University of Florence, Italy. They were maintained in a pathogen-free, temperature-controlled, 12h light and dark cycle environment and were fed ad libitum. Mice were housed in plastics cages (no more than four animals per cage to minimize aggressiveness).
Wild animals	This study does not involve wild animals.
Field-collected samples	This study does not involve samples collected from the field.
Ethics oversight	All animal protocols were approved by the local ethic committee of CeSAL (Centro Stabulazione Animali da Laboratorio) and by the Italian Ministry of Health, in accordance with Italian (D.Lgs. 26/2014) and international (Directive 2010/63/EU) laws and policies.

Note that full information on the approval of the study protocol must also be provided in the manuscript.

Human research participants

Policy information about studies involving human research participants

Population characteristics	Nine pre-treatment patients (F=5, M=4, mean age 80 years) with primary (n=4) or metastatic (n=5) melanomas were used to derive short-term melanoma cultures (Me-14, Me-16, Me-17, Me-25, Me-28, Me-32, Me-41, Me-42 and Me-44).<br>Three patients with brain melanoma metastases: M12 (male, age 75), M15 (female, age 66) and M27 (female, age 44) were used to derived PDXs for scRNA-seq. M12 and M27 had no therapy prior to PDX harvest, M15 had radiation therapy and Ipilimumab prior to PDX harvest.
Recruitment	Entered the study patients with primary or metastatic melanomas performing surgical excision for clinical purposes. Samples were obtained only when there was an excess of tissues after diagnostic and archival procedures. No potential recruitment biases were detected.
Ethics oversight	The use of melanoma samples (Me-14, Me-16, Me-17, Me-25, Me-28, Me-32, Me-41, Me-42 and Me-44) was approved by the Ethic Committee of the University Hospital of Careggi with protocol numbers N. BIO.13.009 and N. BIO.14.026. Tissue acquisition for M12, M15 and M27 melanoma brain metastases was approved by IRB and PDXs development by IACUC.<br>All the subjects gave their written informed consent to participate in the study.

Note that full information on the approval of the study protocol must also be provided in the manuscript.

Flow Cytometry

Plots

Confirm that:

- [x] The axis labels state the marker and fluorochrome used (e.g. CD4-FITC).
- [x] The axis scales are clearly visible. Include numbers along axes only for bottom left plot of group (a 'group' is an analysis of identical markers).
- [ ] All plots are contour plots with outliers or pseudocolor plots.
- [ ] A numerical value for number of cells or percentage (with statistics) is provided.

Methodology

Sample preparation	For apoptosis analysis, cells were serum-starved for 48hrs and apoptosis was measured using Annexin V.<br>For apoptosis analysis, cells were serum-starved for 48hrs and apoptosis was measured using the Annexin V–phycoerythrin/7-AAD apoptosis kit (BD Biosciences, San Diego, CA, USA) according to the manufacturer's protocol. For circulating melanoma cell analysis, 100ul of blood was collected from mice at surgery of the primary tumors by cardiac puncture through a syringe using heparin as anticoagulant. Red blood cells (RBC) were removed by addition of RBC lysis solution (83mg NH_4Cl, 10mg $KHCO_3$, EDTA 5%, ddH_2O to a final volume of 10ml) followed by centrifugation at 1200g for 10min. Pellets were resuspended in 300ul in PBS before flow cytometry.
Instrument	CytoFLEX S Flow Cytometer (Beckman Coulter)
Software	CytExpert Software for apoptosis analysis; FlowJo software for circulating melanoma cells.
Cell population abundance	Sorting was not performed
Gating strategy	For detection of circulating melanoma cells, live cells were gated by FSC-A/SSC-A. GFP+ cells were gated by FIC using both unstained melanoma cells and the blood form healthy mice as negative controls. For analysis of early and late apoptosis, cells were gated by PE (+) / PC.5 (-) (early apoptosis) and PE (+) / PC.5 (+) (late apoptosis).

- [x] Tick this box to confirm that a figure exemplifying the gating strategy is provided in the Supplementary Information.
